# Synthesis of Cyclobutane Analogue 4: Preparation of Purine and Pyrimidine Carbocyclic Nucleoside Derivatives

**DOI:** 10.3390/molecules24183235

**Published:** 2019-09-05

**Authors:** Noha Hasaneen, Abdelaziz Ebead, Muhammad Murtaza Hassan, Hanan Afifi, Howard Hunter, Edward Lee-Ruff, Nadia S. El-Gohary, Azza R. Maarouf, Ali A. El-Emam

**Affiliations:** 1 Department of Chemistry, Faculty of Science, York University, Toronto, ON M3J 1P3, Canada; 2 Chemistry Department, Faculty of Science, Arish University, Arish, Egypt; 3 Basic Science Department, Faculty of Industrial Education, Beni-Suef University, Beni-Suef, Egypt; 4 Department of Medicinal Chemistry, Faculty of Pharmacy, Mansoura University, Mansoura 35516, Egypt

**Keywords:** nucleoside analogs, cyclobutanes, purine, uracil, thymine

## Abstract

The coupling of 2-bromo-3-benzoyloxycyclobutanone with purine under basic conditions produces two regioisomers consisting of the *N-7* and *N-9* alkylated products in equal amounts in their racemic forms. The distribution of the isomers is consistent with the charge delocalization between the *N-7* and *N-9* positions of the purinyl anion. The structural assignments and relative stereochemistry of each regioisomer were based on 1 and 2D NMR techniques. The relative stereochemistry of the *C-2* and *C-3* substituents in each regioisomer was the *trans* orientation consistent with steric factors in the coupling step. The *N-9* regioisomer was reduced with sodium borohydride to give the all *trans* cyclobutanol as the major product in a stereoselective manner. The alcohol was debenzoylated with sodium methoxide in a transesterification step to give the nucleoside analogue. The regioisomeric pyrimidine nucleosides were prepared by Vorbrüggen coupling of the 3-hydroxymethylcyclobutanone triflate with either thymine or uracil followed by stereoselective hydride addition. Regiospecificity of the coupling at the N-1 position was observed and stereoselective reduction to the trans-disubstituted cyclobutanol structure assignments was based on NMR data.

## 1. Introduction

Nucleosides constitute the components of nucleic acids. These are linked by phosphate units in the structures of DNA and RNA. Structural modifications of nucleosides (nucleoside analogs) result in changes to chemical and biological processes involving nucleic acids, resulting in their physiological properties as antiviral and anticancer agents [1,2]. Carbocyclic derivatives in which the ribose ring is replaced by a carbocycle enhances the metabolic stabilities of these potential drugs [3]. The cyclopentenyl derivatives Carbovir [4] and Abacavir [5] developed in the 1990s are some of the early examples of effective antiviral agents. With the observation of potent antiviral activity of the natural occurring Oxetanocin A, [6] the development and commercialization of the synthetic carbocyclic analogues, Cyclobut-A and Cyclobut-G (Lobucavir), as well as related analogs were realized [7,8,9,10,11,12,13].

In view of rapid mutations of viruses becoming resistant to current drug therapies, the quest for new Reverse Transcriptase (RT) inhibitors with structural modifications is an ongoing pursuit. In the current study we report the stereospecific synthesis of cyclobutanols to which a nucleobase is attached to the *C-2* position of the carbocycle, and the *C-3* position by a methylene pendant in a *cis* orientation to the alcohol function. Furthermore, the regiochemistry of both *N-7* and *N-9* isomers of purinonucleosides produced in the coupling reaction was unambiguously assigned.

We have already described the coupling of 6-chloropurine to the *C-2* position and the *C-3* position by a methylene pendant in recent publications [14,15]. In the current study we report on the coupling reactions of the parent purine nucleobase to the *C-2* position of 3-hydroxylmethylcyclobutanol, and the coupling reactions of pyrimidine bases to a methylene bridged *C-3* cyclobutanol. These compounds constitute novel nucleoside analogs with potential biological activities.

## 2. Results and Discussion

### 2.1. Synthesis of Purine Carbocyclic Analogues

#### 2.1.1. N-Alkylation of Purine to Bromocyclobutanone **1**

Direct alkylation of heterocyclic bases (purine and pyrimidine) with alkyl halides is well documented [16,17]. Such an approach also bears some similarity to the reported glycosylation procedure of Robins [18], where the sodium salts of a purine base react with α-halo-sugars to give the corresponding glyconucleosides.

**Scheme 1:**
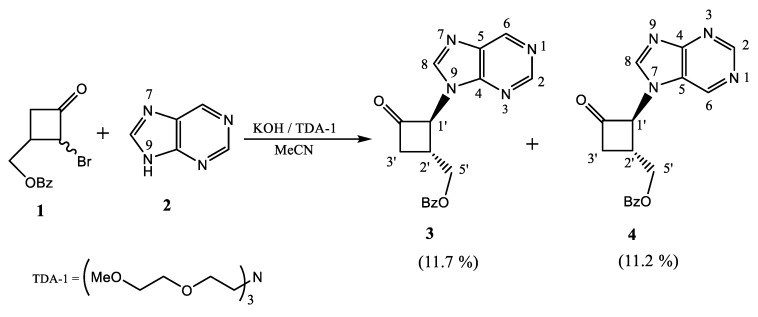
Base assisted coupling of purine with Bromoketone **1**.

Based on these observations, we prepared cyclobutanone derivatives possessing purine as an α-substituent using the following method [19]. The potassium salt of purine (**2**), produced in situ using KOH in the presence of tris(dioxa-3,6-heptyl)amine (TDA-1) in acetonitrile, was treated with α-bromocyclobutanone **1** and resulted in the formation of the *N-9* coupled product **3** in an 11.7% yield as well as the *N-7* regioisomer **4** in an 11.2% yield (Scheme 1). The assignment of the attached site of the purine derivatives **3** and **4** was established using 1D ^1^H- and ^13^C-NMR as well as 2D COSY, HSQC, and HMBC experiments. 

A number of NMR studies have been reported [20,21,22] for the *N-7* and *N-9* purine alkylated derivatives and certain patterns emerge for the ^1^H- and ^13^C-chemical shifts. The signals of H-8, C-1’, C-8, and more significantly C-4 are shifted upfield for the *N-9* isomer relative to the corresponding signals for the *N-7* isomer. By contrast, the C-5 signal for the *N-9* derivatives is deshielded relative to that for the *N-7* isomer due to the larger steric congestion existing in the *N-7* isomer.

**Figure 1:**
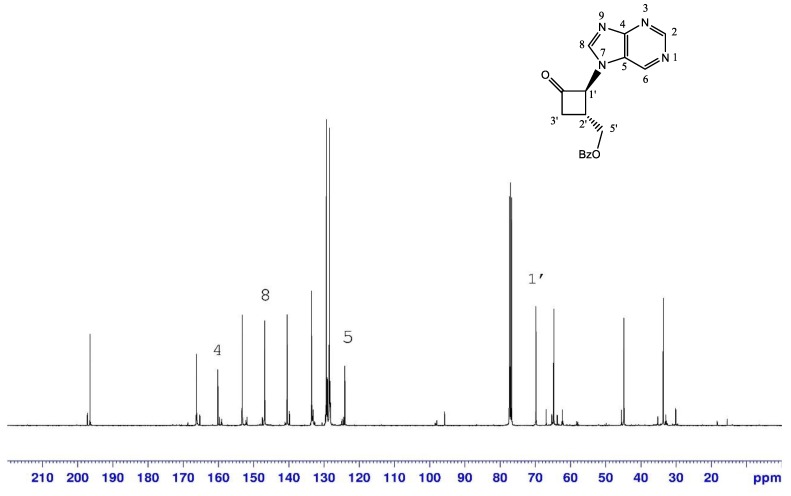
^13^C-NMR spectrum for ketone **4**.

**Figure 2:**
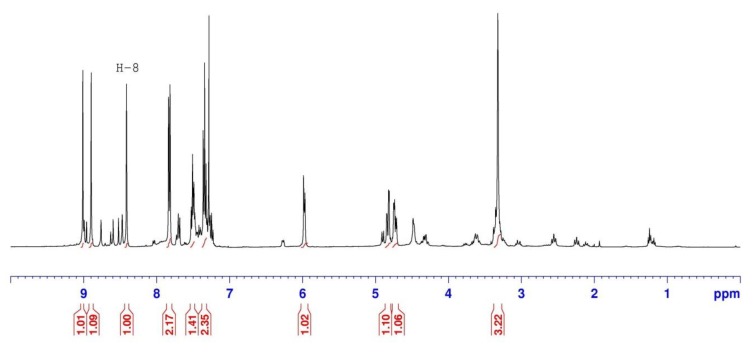
^1^H-NMR spectrum for ketone **4**.

**Figure 3:**
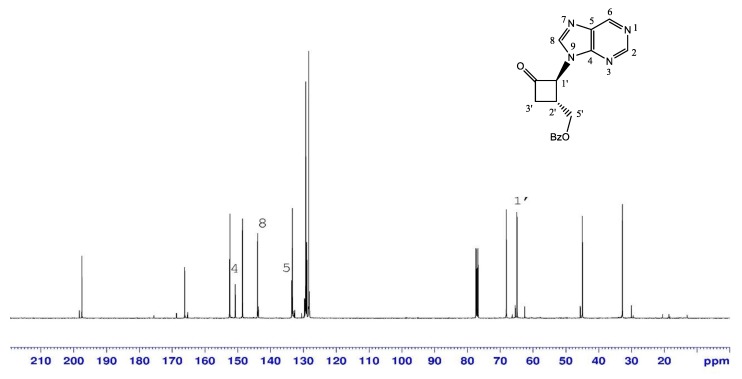
^13^C-NMR spectrum for ketone **3**.

**Figure 4:**
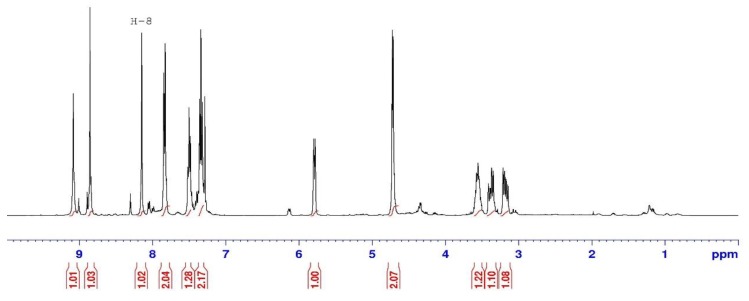
^1^H-NMR spectrum for ketone **3**.

The regiochemistry of **3** and **4** was supported by the larger chemical shift values of H-8 (δ 8.41 ppm), C-1’ (δ 69.8 ppm), C-8 (δ 146.8 ppm), and C-4 (δ 160.2 ppm) signals for the *N-7* isomer **4** (Figure 1 and Figure 2) as compared to the H-8 (δ 8.12 ppm), C-1’ (δ 68.2 ppm), C-8 (δ 144 ppm), and C-4 (δ 150.8 ppm) signals for the *N-9* alkylated derivatives **3** (Figure 3 and Figure 4). This regiochemistry was also supported by the chemical shift of the C-5 signal (δ 133.6 ppm) of **3**, which was shifted downfield relative to the C-5 signal (δ 124.1 ppm) for the *N-7* component **4** (Figure 1 and Figure 2, Table 1). The High Resolution Mass Spectrometry data (HRMS) was consistent with the molecular composition of both **3** and **4**.

**Figure 5:**
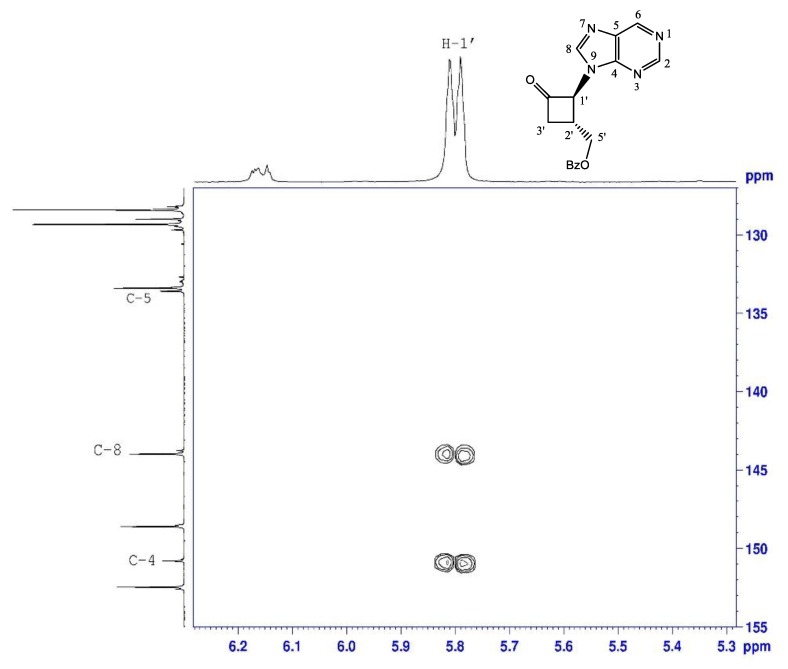
2D-HMBC spectrum for ketone **3**.

**Figure 6:**
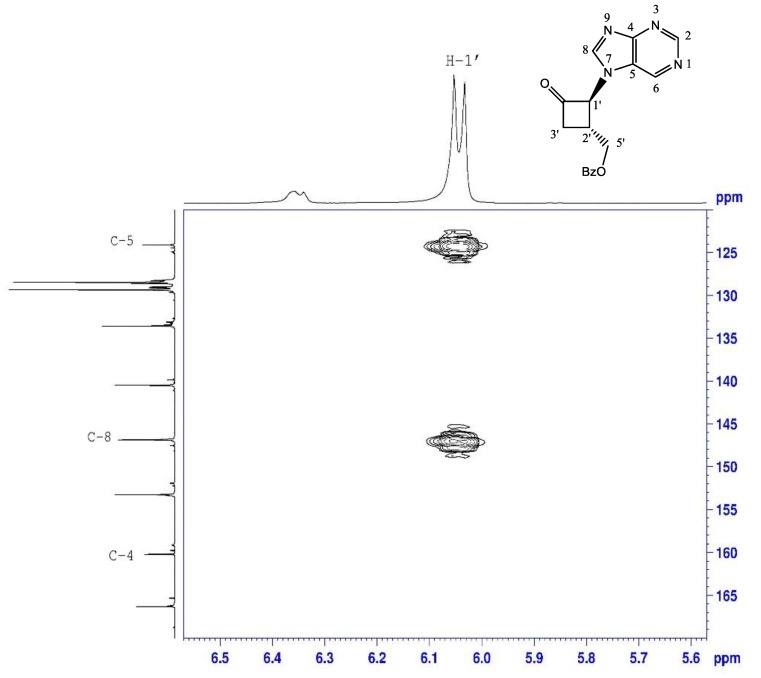
2D-HMBC spectrum for ketone **4**.

The 2D HMBC spectra for **3** and **4** also supported the regiochemical assignments. Correlations between H-1’ and C-8 and C-4 signals were observed with the absence of any correlation with C-5 for the *N-9* isomer **3** (Figure 5). Correlations between H-1’ and C-8 and C-5 signals were observed without any correlation with C-4 for the *N-7* isomer **4** (Figure 6).

**Figure 7:**
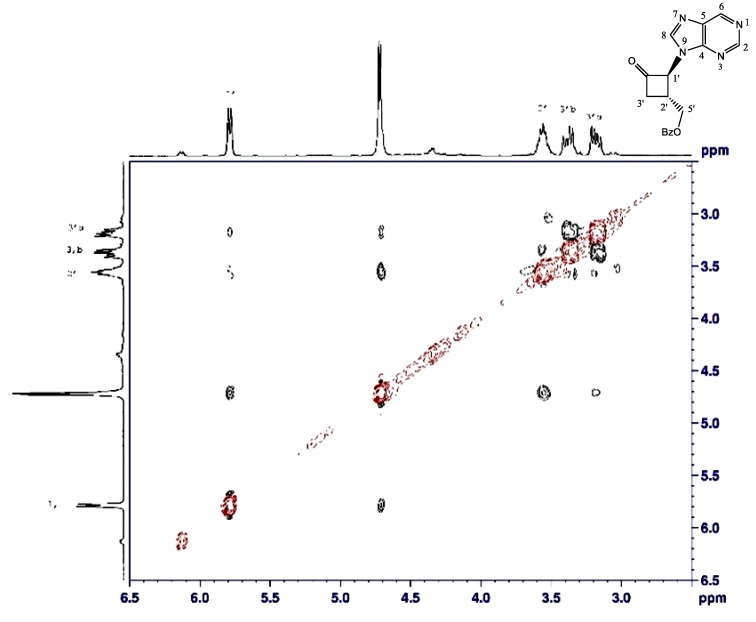
2D-NOESY spectrum for ketone **3**.

**Figure 8:**
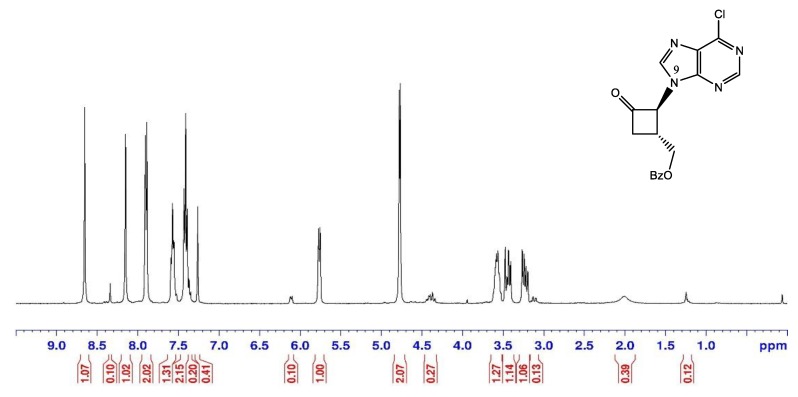
^1^H-NMR spectrum for ketone **5** [14]. Reproduced from thesis of A.E. York University 2011 permission granted by supervisor E.L-R. and York University.

The relative stereochemistry of both the *N-7* and *N-9* alkylated derivatives **3** and **4** were assigned the *trans*-configurations based on 2D NOESY experiments. None of the *cis*-ketone was detected. The NOESY data for **3** (Figure 7) showed correlations between the H-1’ and H-3’a signals, and between the H-2’ and H-3’b signals while no correlation between H-1’ and H-3’b signals, nor between H-2’ and H-3’a signals was observed, indicating that H-1’ and H-2’ are on opposite sides (*trans*-configuration) of the ring. The *trans*-configuration was also supported with the similarity of the ^1^H-NMR spectrum for **3** (Figure 4) with that of *trans*-ketone **5** (Figure 8) the stereochemistry of which was established by X-ray diffraction analysis [14].

**Figure 9:**
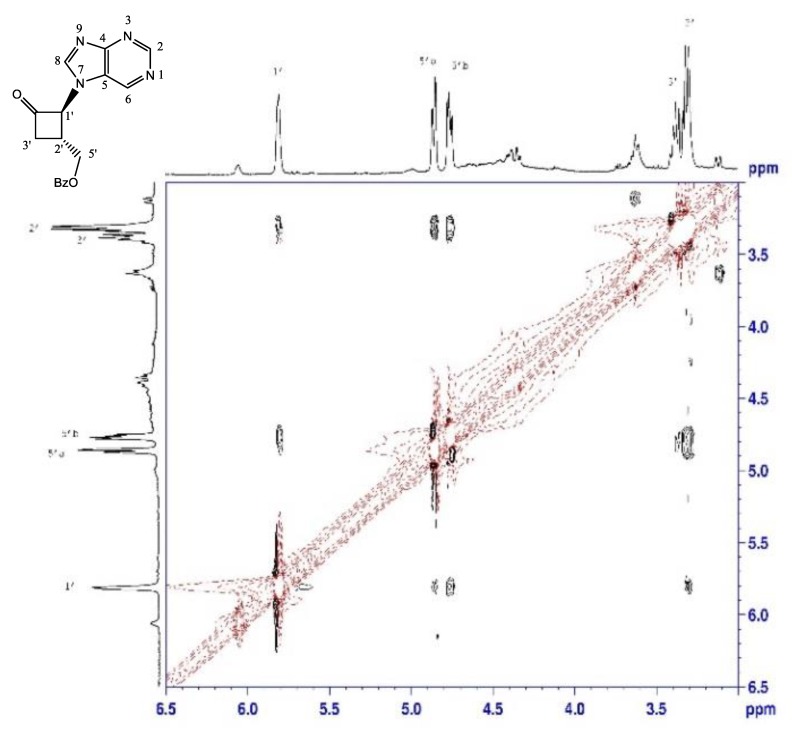
2D-NOESY spectrum for ketone **4**.

The stereochemistry for **4** was determined by the NOESY spectrum (Figure 9), which showed cross-peaks between H-1’ with one of the two H-5’ protons, which indicated their *cis*-configuration and was consistent with the *trans*-configuration for H-1’ and H-2’ protons.

#### 2.1.2. Reduction of Ketone Derivatives

Although we needed to reduce the ketone group as well as deprotect the hydroxymethyl group in ketone **3**, we decided to reduce the ketone group first and then carry out the deprotection step, since the resulting diol diastereomers would be very polar and difficult to separate by chromatography. Therefore, a chemoselective reduction of the ketone group was affected without touching the benzoate group.

**Scheme 2:**
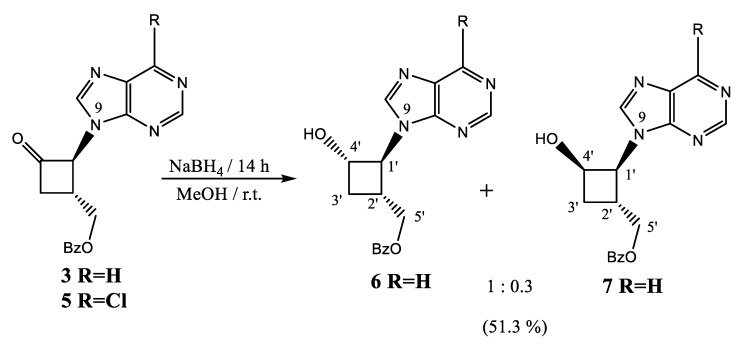
Reduction of Ketone **3**.

Reduction of **3** using NaBH_4_ in MeOH at room temperature for 14 h resulted in the formation of a mixture of *trans*-1’,2’-*trans*-1’,4’-cyclobutanol **6** and *trans*-1’,2’-*cis*-1’,4’-alcohol **7a** in a ratio 1:0.3, respectively (Scheme 2), with a total yield of 51.3%, which could not be separated by either flash chromatography (5–7% methanol in chloroform using gradient elution) or thin layer chromatography (5% methanol in chloroform). No starting material **3** was observed.

The structures of **6** and **7** were supported by the appearance of new downfield proton signals at δ = 4.79 and 5.13 ppm (ratio 1:0.3), respectively. Furthermore, aromatic proton signals for the heterocycle in the δ 9–7.5 range showed the same intensity ratio reflecting the stereoisomeric distribution. The following 2D-NMR analysis was based on the more intense signals of the major alcohol **6**. The absence of the cyclobutanone carbonyl peak in the IR spectrum pointed to complete reaction in the reduction step. HRMS analysis confirmed the molecular composition for **6** and **7**.

**Figure 10:**
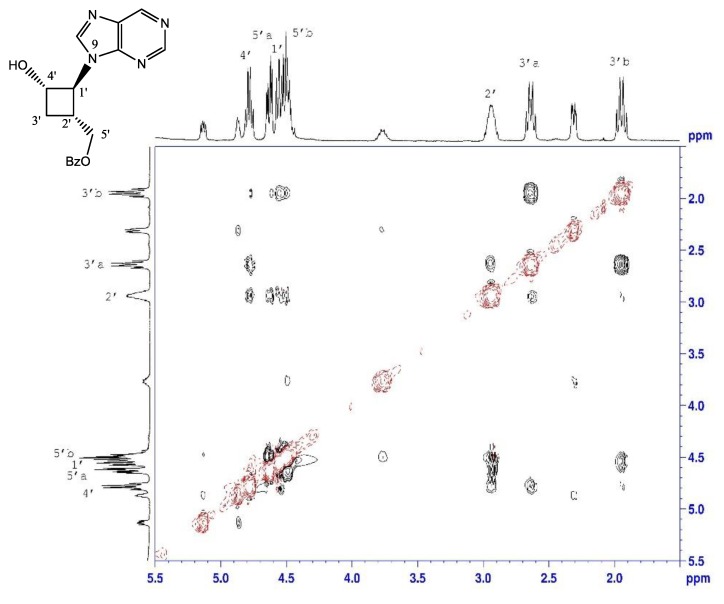
2D-NOESY spectrum for the major signals of alcohol **6**.

The stereochemistry of the major alcohol **6** was assigned based on 1D-^1^H, ^13^C and 2D-COSY, NOESY, and HSQC NMR experiments. The 2D-NOESY spectrum (Figure 10) showed that the H-3’a signal correlated with those for protons H-2’ and H-4’, but not with H-1’, while the H-3’b signal showed a correlation with the H-1’ signal but not with either H-2’ or H-4’ signals, which indicated the *cis* relationship between H-2’ and H-4’ and the *trans* relationship for H-1’. This was consistent with the assigned stereochemistry of **6** to be the *trans*-1’,2’-*trans*-1’,4’-substitution pattern. Since reduction of the ketone produces only two epimers, structure **7** was assigned the other epimer. 

The formation of the *trans*, *trans*-alcohol **6** as the major products in the reduction of the *N-9* ketone **3** was likely due to the directing effect of the proximal lone pair of *N-3* of the purine ring, which coordinated with the boron of the borohydride, leading to the introduction of the hydride ion on the same side of the purine ring, resulting in the hydroxyl group being on the opposite side, i.e., *trans*-configuration.

#### 2.1.3. Debenzoylation of Purine Alcohols

**Scheme 3:**
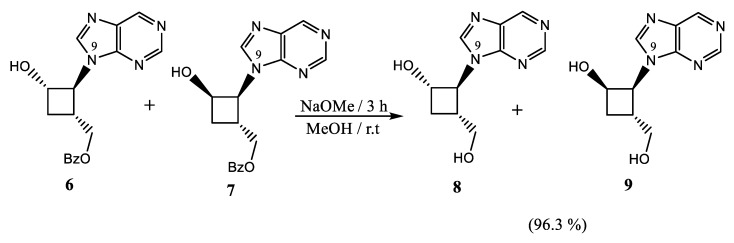
Debenzoylation of esters **6** and **7.**

**Figure 11:**
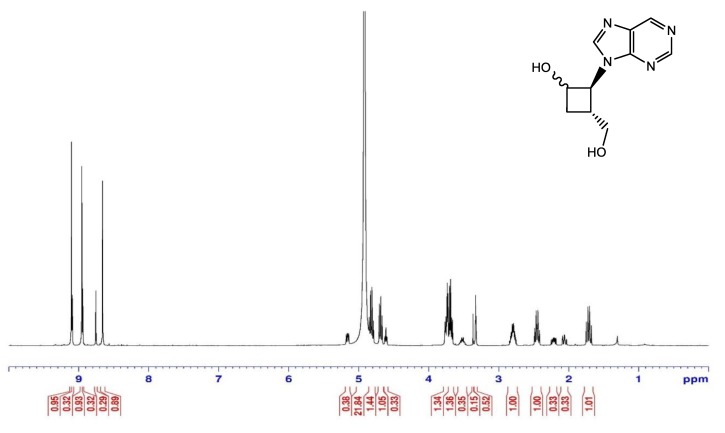
^1^H-NMR spectrum for mixture of purine nucleosides **8** and **9** (1:0.3 ratio).

Debenzoylation of the *N-9* alcohols mixture **6** and **7** using saturated methanolic NaOMe at room temperature for 3 hours afforded a mixture of the purine carbocyclic nucleosides **8** and **9** in an excellent yield (96.3%) in the same 1:0.3 ratio as the original stereoisomeric benzoylated mixture, respectively (Scheme 3). This was indicated from the ^1^H-NMR spectrum (Figure 11). The structures of **8** and **9** were confirmed by the absence of the benzoyl signals in the ^1^H-NMR spectra (Figure 11). The HRMS data for the **8** and **9** mixture were consistent with their molecular formulae. 

Separation of the diol mixture **8** and **9** could not be accomplished by either flash or thin-layer chromatography after numerous attempts. Possible solutions to this problem would include derivatization of either alcohols **6** and **7**, or diols **8** and **9**.

### 2.2. Synthesis of Pyrimidine Carbocyclic Nucleoside Analogues

**Scheme 4:**
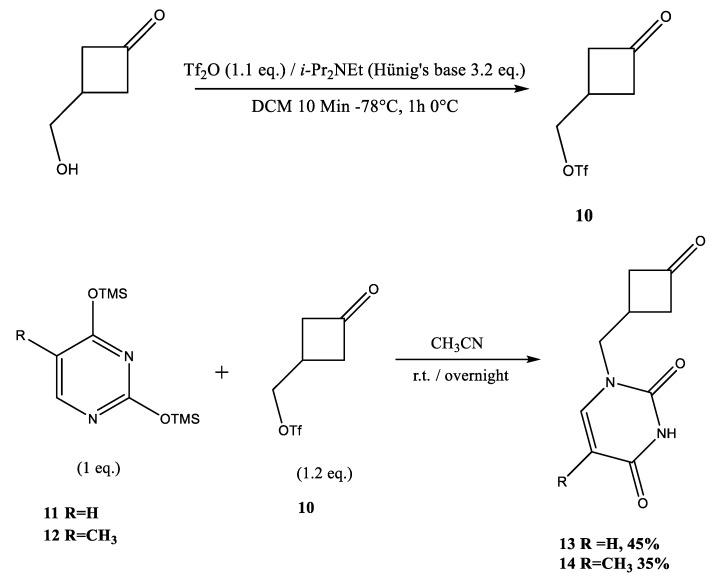
Coupling of ketone **10** with thymine and uracil.

We reported recently on the 6-chloropurine *N-*alkylation to 3-hydroxymethylcyclobutanone triflate **10** [15]. In order to complete the series of these related nucleoside analogs we undertook to couple the pyrimidine nucleobases thymine and uracil with ketone **10**. However, unlike 6-chloropurine, the attempted coupling under basic conditions through the anions did not lead to any meaningful yields for the coupling products. The lack of coupling of the pyrimidines as compared to 6-chloropurine under basic conditions may be due to the lower pK_a_ for the N–H group in the pyrimidine nucleobases. Thus, we resorted to the classical Silyl–Hilbert–Johnson (or Vorbruggen coupling) reaction [23]. This protocol entails the use of silyl protected nucleobases in the coupling with a substrate possessing a labile leaving group under Lewis acid conditions. The specific silyl protected nucleobases **11** and **12** were prepared under standard conditions (Scheme 4) [24].

The coupling of **11** and **12** to **13** and **14**, respectively, was accomplished using 3-hydroxymethylcyclobutanone triflate **10** generated in situ prepared from 3-hydroxycyclobutanone and reacting with the silylated nucleobase **11** or **12** giving **13** and **14**, in yields of 45 and 35%, respectively, as crystalline solids. 

**Scheme 5:**
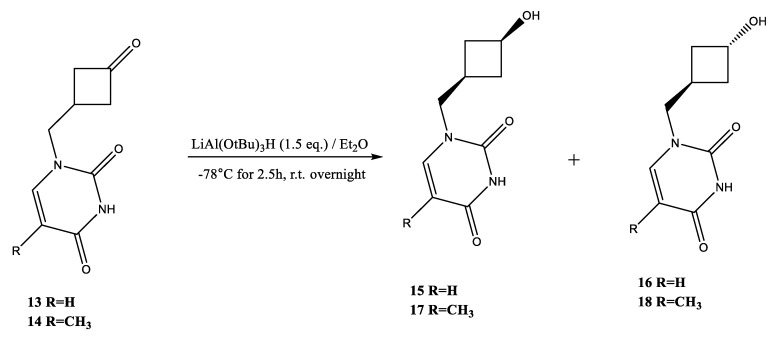
Reduction of ketones **13** and **14.**

The final reduction step was carried out with the bulky hydride donor, lithium aluminum *t*-butoxide. Unlike the corresponding 6-chloropurine analog, which gave a single stereoisomer, pyrimidine ketones gave stereoisomeric mixtures of **15** and **16** for **13**, and **17** and **18** for **14** (Scheme 5). The *cis* stereoisomers **15** and **17** still predominated by at least 10:1 in each case as expected from hydride delivery to the less hindered side of the cyclobutanone ring. The lower stereoselectivity in the reduction of **13** and **14** is attributed to the smaller steric bulk of pyrimidine as compared to 6-chloropurine.

The regio- and stereochemistry for **15** and **17** were assigned by the following 2D NMR analyses as seen by their NMR spectra (see Supplementary Materials).

**Scheme 6:**
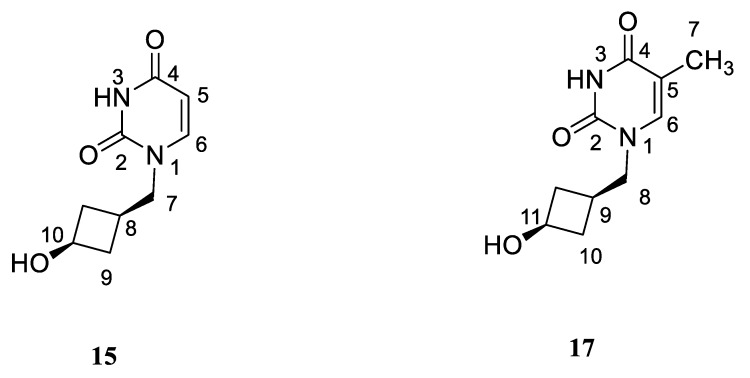
Structure numbering for 2D NMR analysis.

For **15** the vinyl protons from the uracil ring were readily identified at 5.623 and 7.551 ppm for positions 5 and 6, respectively (see Scheme 6 for position numbering). Proton 5 showed ^1^H–^13^C multiple bond correlations to carbons 4 and 6 at 167.25 and 147.90 ppm, respectively, in the 2D ^1^H–^13^C HMBC spectrum. Proton 6 showed ^1^H–^13^C multiple bond correlations to carbon-2 at 153.38 ppm as well as to a methylene carbon at 54.68 ppm. This carbon would have corresponded to C-7 and the correlation would only have been possible if the alkyl substitution was at nitrogen 1. Likewise, the 2D ^1^H–^13^C HMBC also showed correlations from protons 7 to carbons 2, 6 (at 147.90 ppm), 8 (at 26.78 ppm), and 9 (at 38.26 ppm) and provided convincing evidence of alkyl substitution at the 1 position. Using the 2D ^1^H–^1^H COSY spectrum, protons 8, 9, and 10 could be identified. Position 9 had identical shifts with prochiral protons. Protons on one face of the cyclobutane ring had a shift of 1.650 ppm, while the opposite face had a shift of 2.357 ppm. 

The 2D ^1^H–^1^H NOESY also indicated that protons 6 and 7 were close together in space. In addition, there was an intense correlation between proton 7 and proton 9 at 1.650 ppm, suggesting that this interaction was between protons on the same side of the cyclobutane ring. Conversely, the protons at positions 8 and 10 had more intense correlations to proton 9 at 2.357 ppm than they did to proton 9 at 1.650. This evidence supports the structure with protons 8 and 10 both on the same side of the cyclobutane ring.

For **17** the aromatic and methyl protons from the thymine ring were readily identified at 7.401 and 1.855 ppm, respectively. The methyl proton at 1.855 ppm showed ^1^H–^13^C multiple bond correlations to carbons 4, 5, and 6 at 167.36, 111.45, and 143.79 ppm, respectively, in the 2D ^1^H–^13^C HMBC spectrum. In addition, the proton at 7.401 ppm (6) showed ^1^H–^13^C multiple bond correlations to carbon 2 at 153.52 ppm. Proton 6 also showed ^1^H–^13^C multiple bond correlations to a methylene carbon at 54.4 ppm. This carbon would have corresponded to 8 and the correlation would have only been possible if the alkyl substitution was at nitrogen 1. Likewise, the 2D ^1^H–^13^C HMBC also showed correlations from proton 8 to carbons 2, 5, and 6 and provided convincing evidence of alkyl substitution at the 1 position. Using the 2D ^1^H–^1^H COSY spectrum, protons 9, 10, and 11 could be identified. Position 10 had identical shifts with prochiral protons. Protons on one face of the cyclobutane ring had a shift of 1.653 ppm, while the opposite face had a shift of 2.347 ppm. 

The 2D ^1^H–^1^H NOESY also indicated that protons 6 and 8 were close by in space. In addition, there was an intense correlation between protons 8 and 10 at 1.653 ppm, suggesting that this interaction was between protons on the same side of the cyclobutane ring. Conversely, the protons at positions 9 and 11 had more intense correlations to proton 10 at 2.347 ppm than they did to protons 10 at 1.653. This evidence supports the structure with protons 9 and 11 both on the same side of the cyclobutane ring.

## 3. Summary and Conclusions

Two classes of cyclobutane nucleoside analogs were prepared. Parent purine was coupled with 2-bromo-3-hydroxymethylcyclobutanone to give both the *N-7* and *N-9* regioisomers by way of the purinyl anion. The anion has the negative charge delocalized at both *N-7* and *N-9* centers. The *N-9* coupled ketone was structurally elaborated to the diol in two steps in order for the attempted separation of the stereoisomeric mixture in the initial reduction with NaBH_4_ in which the *trans, trans*-alcohol predominates in a 3:1 ratio. This mixture was debenzoylated with sodium methoxide to give the diol mixture in the same ratio.

The other class of cyclobutane analogs, where the nucleobase is bonded to the cyclobutane ring via a methylene pendant at the *C-3* position, was accomplished by treating 3-hydroxymethylcyclobutanone triflate and coupling with the silylated uracil and thymine under Vorbruggen conditions. Only the *N-1* coupled products were observed for both uracil and thymine. The ketones were stereoselectively reduced to the cyclobutanols with the bulky hydride donor, sodium *t*-butoxide, to give the *cis*-alcohols in both cases. 

In view of the reported antiviral activities of the carbocyclic analogs of oxetanocin, these novel structurally related compounds may be good candidates to possess biological activity. It should be noted that the cyclobutane purine analogs were prepared in their racemic modifications from racemic **1**, the starting precursor. Once biological activity is established the enantiomeric forms will be resolved in order to identify the specifically active component. The pyrimidine analogs possess a plane of symmetry and hence exist as single stereoisomers.

## 4. Experimental

Melting points (mp) were obtained on a Fisher–Johns melting point apparatus (Thermo Fisher Scientific, Carlsbad, CA, USA) and were uncorrected. Fourier transform infrared spectra were recorded on a Genesis II Mattson 3000 FT-IR spectrometer (Mattson Technology, Fremont, CA, USA) as thin films and on a Nicolet 380 FT-IR spectrometer (Nicolet Instrument Inc., Madison, WI, USA)as a solid disk. UV spectra were determined using an Ultrospec 4300 pro UV spectrometer (GE, Boston, MA, USA). HRMS analysis was performed at Queen’s University (Kingston, ON, Canada). LC–MS analyses were obtained on a Waters 2695LC-Quatro Ultima MS instrument (Waters Corporation, Milford, MA, USA). Elemental analysis was performed at Guelph Chemical Laboratories Limited (Guelph, ON, Canada).The 1D and 2D-NMR analyses were recorded on Bruker AV 400 (400.13 MHz) and DRX 600 (599.86 MHz) NMR spectrometers (Bruker Biospin, Billerica, MA, USA). Data for ^1^H-NMR were referenced relative to the solvent residual proton signals, CDCl_3_ (δ 7.27), DMSO-d_6_ (δ 2.50), and CD_3_OD (δ 3.31). Data for ^13^C-NMR were referenced relative to the solvent residual carbon signals, CDCl_3_ (δ 77.16), DMSO-d_6_ (δ 39.52), and CD_3_OD (δ 49.00). The 1D-NMR chemical shifts were quoted in delta (δ) values in parts per million (ppm) downfield from TMS (δ = 0). Tabulation of 1D-NMR data was reported in the following order: chemical shifts (δ in ppm), multiplicity (s = singlet, d = doublet, t = triplet, dxd = doublet of doublets, m = multiplet), coupling constant (*J* in Hz), and integration (number of protons).

### 4.1. N-Alkylation of Purine

Potassium hydroxide (0.61 g, 10.8 mmol) and TDA-1 (0.29 g, 0.9 mmol) were added to purine (1.08 g, 9 mmol) in 100 mL of acetonitrile and the mixture was stirred overnight at room temperature. A solution of α-bromocyclobutanone **1** (3.06 g, 10.8 mmol) in acetonitrile (15 mL) was added dropwise and the mixture was stirred overnight at room temperature. Insoluble material was filtered off and the solvent was evaporated under vacuum. The residue was purified by chromatography (2% methanol in chloroform) to afford the *trans*-*N-9*-alkylated derivative **3** (0.34 g (11.7%) as well as 0.33 g (11.2%) of the *trans*-*N-7*-alkylated derivative **4** as an oil.

*Trans-(3-oxo-2-(9H-purin-9-yl)cyclobutyl)methyl benzoate* (**3**). ^1^H-NMR (400 MHz, CDCl_3_), δ 9.06 (s, 1H), 8.83 (s, 1H), 8.12 (s, 1H), 7.81 (d, *J* = 7.6 Hz, 2H), 7.48 (t, *J* = 6.8 Hz, 1H), 7.32 (t, *J* = 7.6 Hz, 2H), 5.77 (d, *J* = 8.0 Hz, 1H), 4.70 (d, *J* = 5.6 Hz, 2H), 3.54 (m, 1H), 3.35 (m, 1H), 3.16 (m, 1H); ^13^C-NMR (400 MHz, CDCl_3_), δ 197.5, 166.2, 152.5, 150.8, 148.6, 144.0, 133.6, 133.4, 129.3, 129.0, 128.4, 68.2, 64.9, 45.0, 32.8; HRMS (EI) *m/z* calculated for C_17_H_14_N_4_O_3_ (M^+^) 322.1066, found 322.1057.

*Trans-(3-oxo-2-(7H-purin-7-yl)cyclobutyl)methyl benzoate* (**4**). ^1^H-NMR (400 MHz, CDCl_3_), δ 9.02 (s,1H), 8.90 (s, 1H), 8.41 (s, 1H), 7.83 (d, *J* = 7.6 Hz, 2H), 7.51 (t, *J* = 7.4 Hz, 1H), 7.34 (t, *J* = 7.8 Hz, 2H), 5.97 (d, *J* = 7.2 Hz,1H), 4.83 (m, 1H), 4.74 (m, 1H), 3.33 (m, 3H); ^13^C-NMR (400 MHz, CDCl_3_), δ 196.5, 166.3, 160.2, 153.3, 148.6, 140.5, 133.6, 129.4, 128.6, 128.5, 124.1, 69.8, 64.8, 44.8, 33.6; HRMS (EI) *m/z* calculated for C_17_H_14_N_4_O_3_ (M^+^) 322.1066, found 322.1053.

*Benzoic acid 2-(6-chloro-purin-9-yl)-3-oxo-cyclobutylmethyl ester* (**5**) [14]. ^1^H-NMR (400 MHz, CDCl3), *δ* 8.65 (s, 1H), 8.15 (s, 1H), 7.90 (d, *J* = 7.6 Hz, 2H), 7.57 (t, *J* = 6.8 Hz, 1H), 7.41 (t, *J* = 7.8 Hz, 2H), 5.76 (d, *J* = 8.0 Hz, 1H), 4.77 (d, *J* = 5.2Hz, 2H), 3.56 (m, 1H), 3.44 (m, 1H), 3.23 (dxd, *J* = 8.8, 18.0Hz, 1H); (Figure 8).

### 4.2. Reduction of N-9-Purine Ketone ***3***

To a solution of *trans*-N9-ketone **3** (0.499 g, 1.55 mmol) in methanol (30 mL), 0.059 g (1.55 mmol) of sodium borohydride (NaBH_4_) was added and the mixture was stirred at r.t for 14 h. Saturated aqueous solution of NH_4_Cl (35 mL) was added and stirred for 10 min. The solvent was removed under vacuum and the organic mixture was extracted from the residue using 25% MeOH in CHCl_3_. The solvent was evaporated, and the residue was purified by column chromatography (5–7% methanol in chloroform using gradient elution) to give 0.258 g (51.3%) of a mixture of alcohols **6** and **7** as a gum with a ratio 1:0.3, respectively (based on ^1^H-NMR spectrum).

### 4.3. Major Alcohol

*Trans, trans-3-hydroxy-2-(9H-purin-9-yl)cyclobutyl)methyl benzoate* (**6**). ^1^H-NMR (400 MHz, CDCl_3_), δ 9.04 (s, 1H), 8.86 (s, 1H), 8.21 (s, 1H), 7.89 (d, *J* = 7.6 Hz, 2H), 7.55 (t, *J* = 7.2 Hz, 1H), 7.40 (t, *J* = 7.8 Hz, 2H), 4.79 (dxd, *J* = 8.0, 15.6 Hz, 1H), 4.62 (dxd, *J* = 5.0, 11.8 Hz, 1H), 4.55 (t, *J* = 8.0 Hz, 1H), 4.49 (dxd, *J* = 6.8, 11.6 Hz, 1H); 2.94, (m, 1H), 2.63 (dxd, *J* = 8.2, 19.0 Hz, 1H), 1.94 (dxd, *J* = 10.4, 19.6 Hz, 1H); ^13^C-NMR (400 MHz, CDCl_3_), δ 166.3, 151.7, 151.5, 148.0, 144.3, 133.9, 133.3, 129.3, 129.2, 128.4, 67.9, 65.5, 61.7, 31.7, 28.2; HRMS (for alcohol mixture) (EI) *m/z* calculated for C_17_H_16_N_4_O_3_ (M^+^) 324.1222, found 324.1212.

### 4.4. Minor Alcohol

*Cis, trans-3-hydroxy-2-(9H-purin-9-yl)cyclobutyl)methyl benzoate* (**7**). ^1^H-NMR (400 MHz, CDCl_3_), δ 8.90 (s, 1H), 8.79 (s, 1H), 8.52 (s, 1H), 7.79 (d, *J* = 7.6 Hz, 2H), 7.52 (t, *J* = 7.6 Hz, 1H), 7.35 (t, *J* = 8.0 Hz, 2H), 5.13 (dxd, *J* = 5.4, 9.0 Hz, 1H), 4.86 (m, 1H), 4.47 (m, 2H), 3.76 (m, 1H), 2.30 (dxd, *J* = 3.4, 9.4 Hz, 2H); ^13^C-NMR (400 MHz, CDCl_3_), δ 166.2, 151.5, 151.0, 166.7, 166.2, 133.9, 133.2, 129.2, 128.4, 128.3, 68.4, 65.4, 54.0, 39.5, 29.0.

### 4.5. Deprotection of Purine Alcohols ***6*** and ***7***

To 0.16 g (0.49 mmol) of *N-9*-alcohol mixture **6** and **7**, 4 mL of saturated solution of NaOMe in methanol was added and the mixture was stirred at room temperature for 3 h. Saturated aqueous solution (12 mL) of NaHCO_3_ was added and the mixture was stirred for 15 min. The solvent was evaporated under vacuum and the organic mixture was extracted from the residue using 5% methanol in chloroform. The solvent was removed to obtain 0.105 g (96.3%) of a mixture of purine carbocyclic nucleosides **9** and **10** as a gum in a ratio 1:0.3, respectively.

*Trans, trans-3-hydroxymethyl-2-purin-9-yl-cyclobutanol* (**8**). ^1^H-NMR (400 MHz, CD_3_OD), δ 9.08 (s, 1H), 8.93 (s, 1H), 8.66 (s, 1H), 4.82 (dxd, *J* = 8.0, 15.6 Hz, 1H), 4.69 (m, 1H), 3.72 (m, 2H), 2.80 (m, 1H); 2.44, (m, 1H), 1.72 (dxd, *J* = 10.2, 19.0 Hz, 1H); ^13^C-NMR (400 MHz, CD_3_OD), δ 151.6, 151.5, 147.2, 146.0, 133.8, 67.7, 62.1, 60.4, 34.7, 27.5; HRMS (for **172** and **173** mixture) (EI) *m/z* calculated for C_10_H_12_N_4_O_2_ (M^+^) 220.0960, found 220.0969.

*Cis, trans-3-hydroxymethyl-2-purin-9-yl-cyclobutanol* (**9**). ^1^H-NMR (400 MHz, CD_3_OD), δ 9.06 (s, 1H), 8.91 (s, 1H), 8.76 (s, 1H), 5.16 (dxd, *J* = 5.4, 8.6 Hz, 1H), 4.62 (t, *J* = 5.4 Hz, 1H), 3.72 (m, 2H), 3.52 (m, 1H), 2.22 (m, 1H), 2.06 (m, 1H).; ^13^C-NMR (400 MHz, CD_3_OD), δ 151.6, 151.3, 146.8, 146.7, 133.2, 67.0, 62.4, 53.1, 41.4, 27.8.

### 4.6. Preparation of Ketone ***13***

In round bottom flask, 3-(hydroxymethyl)cyclobutan-1-one (0.998 mmol, 100 mg) in 10 mL dry methylene chloride was added and cooled to −78 °C. Hünig’s base (3.1936 mmol, 0.556 mL) was added and after 5 min this was followed by addition of trifluoromethane sulfonic anhydride (1.098 mmol, 0.185 mL). The reaction mixture was stirred at −78 °C for 10 min then warmed to 0 °C and continued for 1 h. To this flask was added silylated uracil **11** (0.8316 mmol, 213.28 mg) in 10 mL dry acetonitrile and stirred overnight at room temperature. This was then followed by evaporation of the solvents under vacuum and purification by column chromatography packed with silica gel using 3%MeOH/CHCl_3_. We recovered 83 mg (43%) of a white solid, melting point 203–206 °C, Rf: 0.357, with 5%MeOH/CHCl_3__._

The IR spectrum can be found in the Supplementary Materials.

### 4.7. Preparation of Ketone ***14***

In round bottom flask, 3-(hydroxymethyl)cyclobutan-1-one (1.997 mmol, 200 mg) in 20 mL dry methylene chloride was added and cooled to −78 °C. To this solution was added (6.39 mmol, 1.112 mL) Hünig’s base, and after 5 min trifluoromethane sulfonic anhydride (2.1967 mmol, 0.37 ml) was added. The mixture was stirred at −78 °C for 10 min then warmed to 0 °C and continued for 1 h. To this flask was added silylated thymine **12** (1.664 mmol, 450 mg) in 20 mL dry acetonitrile, and stirring continued overnight at room temperature. The solvents were evaporated under vacuum and purified using column chromatography packed with silica gel using 20% hexane/ethyl acetate as eluent. After further purification using preparative TLC with 7% MeOH/DCM, 121.3 mg (35%) of a white solid was obtained, melting point 199–204 °C, Rf: 0.277, with 3% MeOH/DCM.

The IR spectrum can be found in the Supplementary Materials.

### 4.8. Preparation of Alcohol ***15***

In a round bottom flask, ketone **13** (0.257 mmol, 50 mg) in 10 mL dry ether was placed and cooled to −78 °C, followed by addition of LiAl(OtBu)_3_H 1 M in dry THF (0.386 mL, 0.386 mmol) dropwise and stirred for 2.5 h at −78 °C, then overnight at room temperature. The mixture was quenched with saturated aqueous ammonium chloride solution and stirred for 20 minutes. Evaporation of the solvents under vacuum and extraction with 25% MeOH/DCM, followed by drying with anhydrous MgSO_4_, and evaporation under vacuum gave a mixture that was further purified using preparative TLC with 8% MeOH/DCM. A 43 mg quantity of a colorless viscous oil (43 mg) was obtained; yield 85%, Rf: 0.2 on TLC using 5% MeOH/DCM as eluent.

HRMS: Calculated *m/e* 197.77052; Found 197.07747.

Details of the NMR data can be found in the Supplementary Materials.

### 4.9. Preparation of Alcohol ***17***

In a round bottom flask, ketone **14** (0.24 mmol, 50 mg) in 10 mL dry ether was placed and cooled to −78 °C, followed by addition of LiAl(OtBu)_3_H 1 M in dry THF (0.36 mL, 0.36 mmol) drop wise and stirred for 2.5 h at −78 °C then overnight at room temperature. The mixture was quenched with saturated aqueous ammonium chloride solution and stirred for 20 minutes. Evaporation of the solvents under vacuum, followed by extraction of the product with 25% MeOH/DCM, drying with anhydrous MgSO_4_, and evaporation under vacuum gave a mixture that was further purified using preparative TLC with 7.5% MeOH/DCM. A 33 mg quantity of a white solid was obtained; yield 60.6%, Rf: 0.138 on TLC with 3% MeOH/DCM as eluent.

HRMS: Calculated *m/e* 209.09317; Found 209.09289.

Details of the NMR data can be found in the Supplementary Materials.

## Figures and Tables

**Table 1:** ^1^H- and ^13^C-NMR chemical shifts (ppm) for **3** and **4** ketones.

	*N-9*-ketone 3	*N-7*-ketone 4
H-8	8.12	8.41
C-1’	68.2	69.8
C-4	150.8	160.2
C-5	133.6	124.1
C-8	144	146.8

## Supplementary Materials

The following are available online, the IR spectra of Ketones **13** and **14**, the 1D and 2D NMR data for alcohols **15** and **17**.
