# Supplementary material for: Synthesis of Cyclobutane Analogue 4: Preparation of Purine and Pyrimidine Carbocyclic Nucleoside Derivatives
Source: Molecules. 2019 Sep 5;24(18):3235. doi: 10.3390/molecules24183235 (PMC6767184; doi:10.3390/molecules24183235)
Supplement: Supplementary file 1 [file molecules-24-03235-s001.zip › molecules-576798-supplementary.docx]

*Supplementary Material*

Synthesis of Cyclobutane Analogue 4: Preparation of Purine and Pyrimidine Carbocyclic Nucleoside Derivatives

Noha Hasaneen ^1^, Abdelaziz Ebead ^2^, Murtaza Hassan ^1^, Hanan Afifi ^3^, Howard Hunter ^1^, Edward Lee-Ruff ^1,^*, Nadia S. El-Gohary ^4^, Azza R. Maarouf ^4^ and Ali A. El-Emam ^4^

^1^ Department of Chemistry, Faculty of Science, York University, Toronto, Ontario M3J 1P3, Canada

^2^ Chemistry Department, Faculty of Science, Arish University, Arish, Egypt

^3^ Basic Science Department, Faculty of Industrial Education, Beni‐Suef University, Beni‐Suef, Egypt

^4^ Department of Medicinal Chemistry, Faculty of Pharmacy, Mansoura University, Mansoura 35516, Egypt

***** Correspondence: leeruff@yorku.ca; Tel.: 416-736-5443

**IR spectrum of 13**


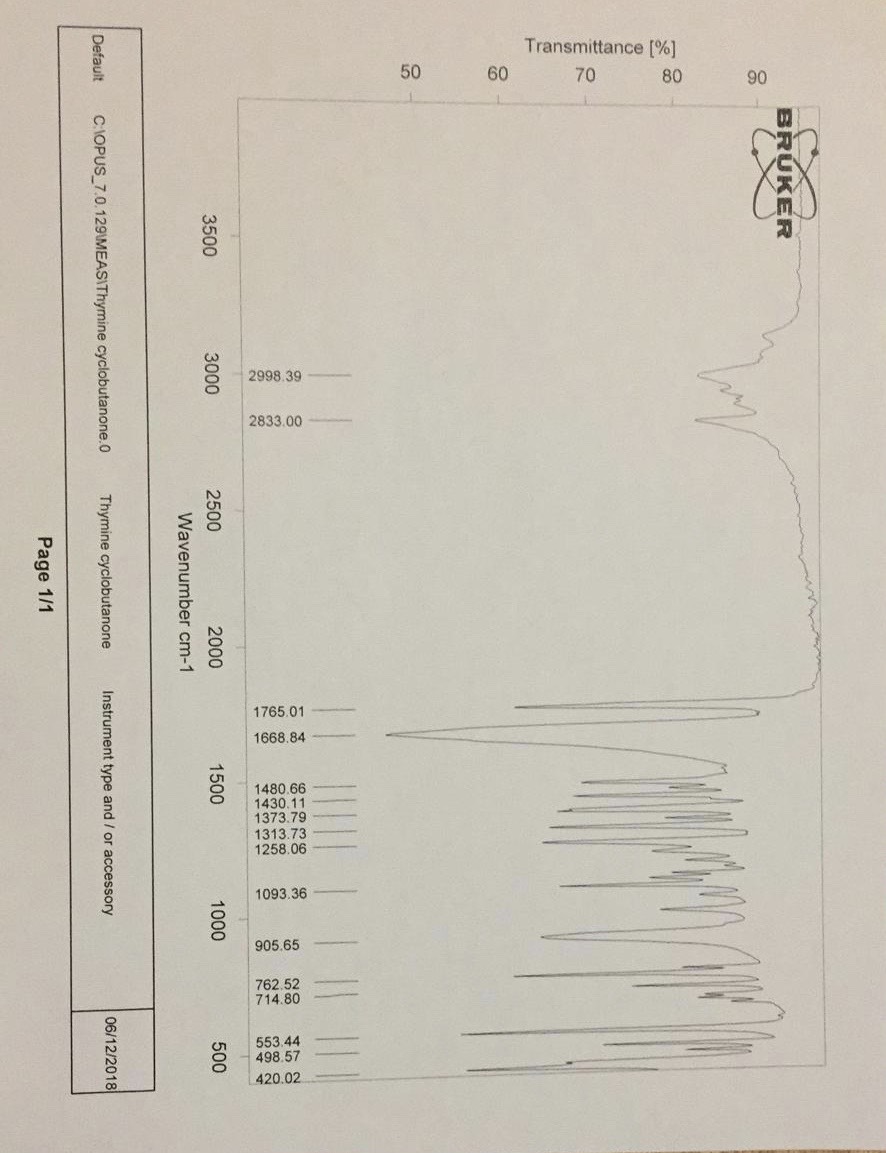


**IR spectrum of 14**


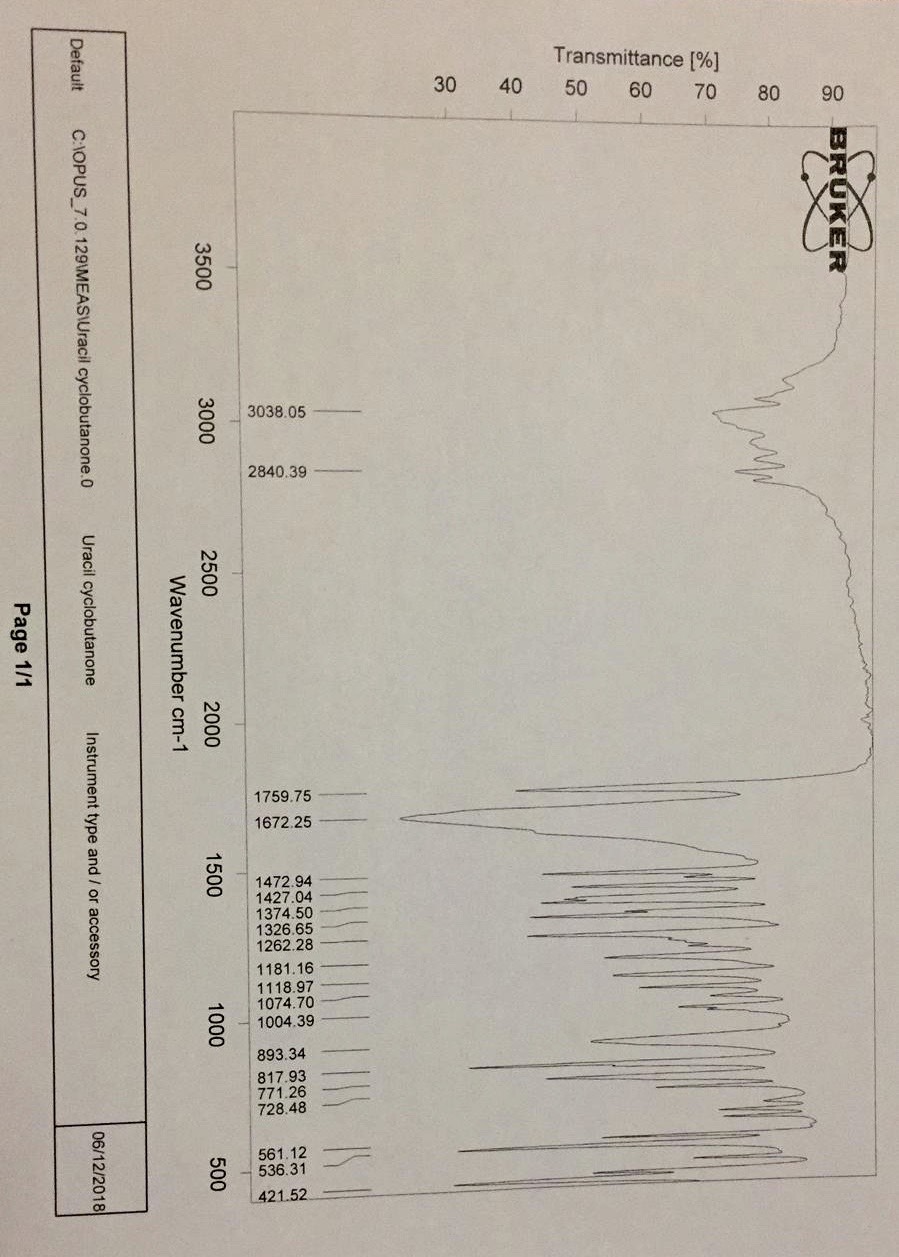


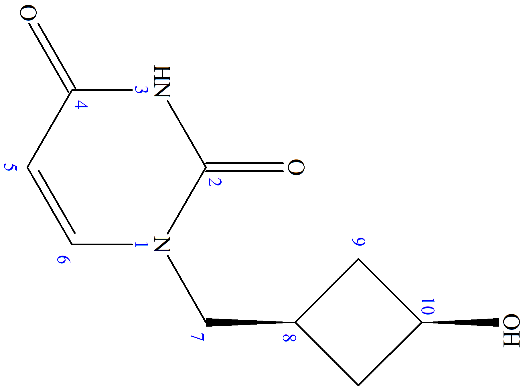

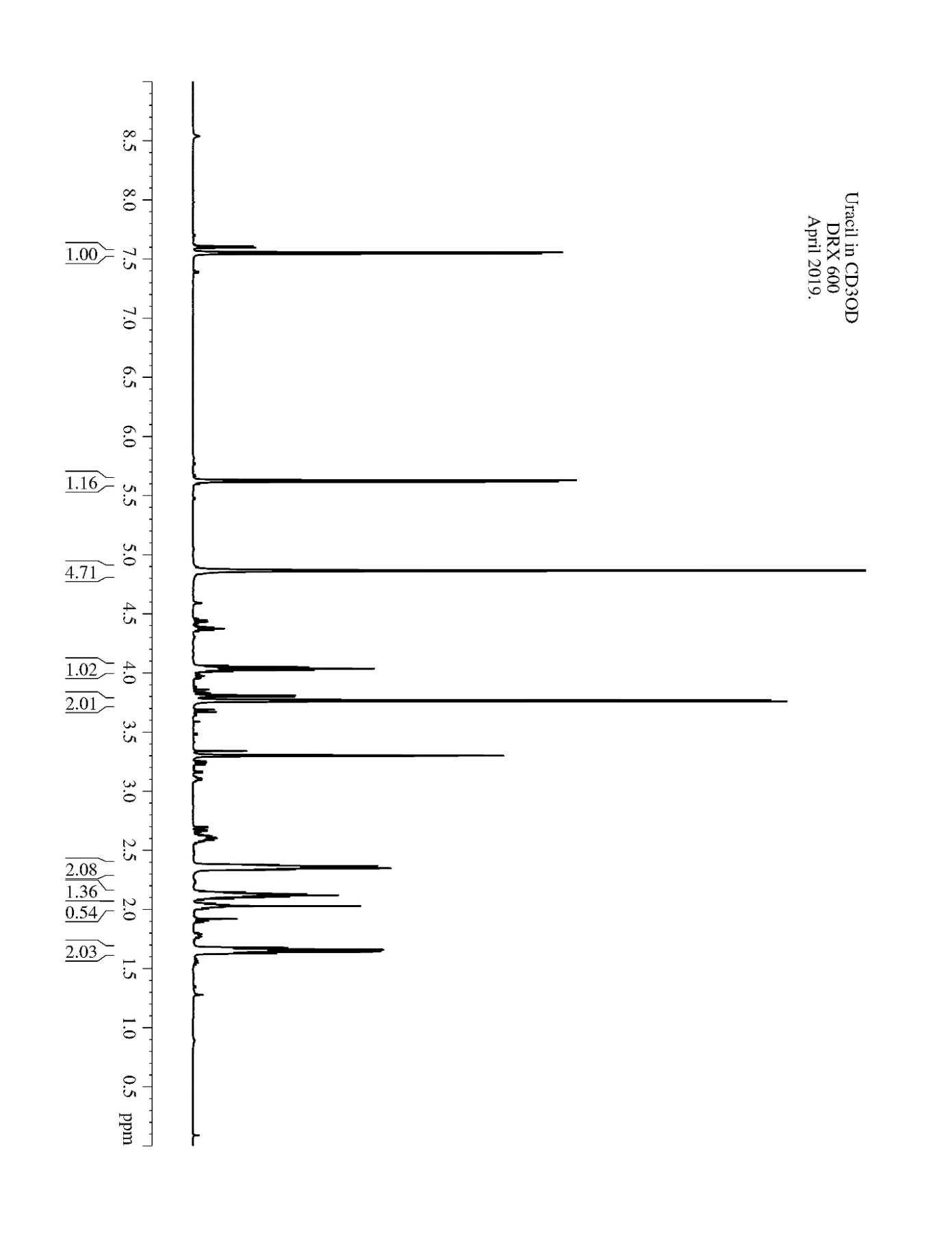


8

6

7

10

9

9

5

DRX 600 1D ^1^H NMR Spectra of Uracil **15** in CD_3_OD


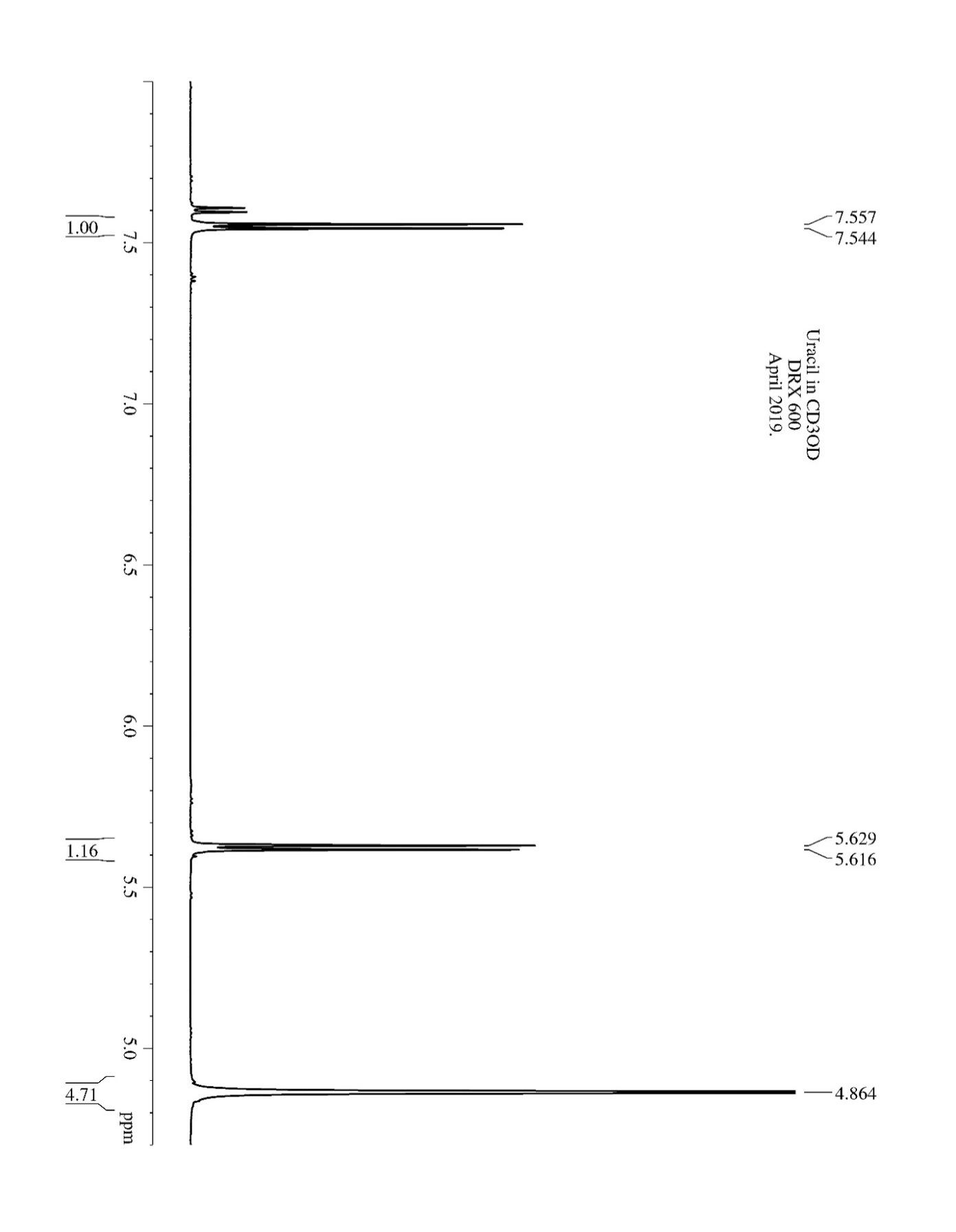


DRX 600 1D ^1^H NMR Spectra of Uracil Sample **15** in CD_3_OD


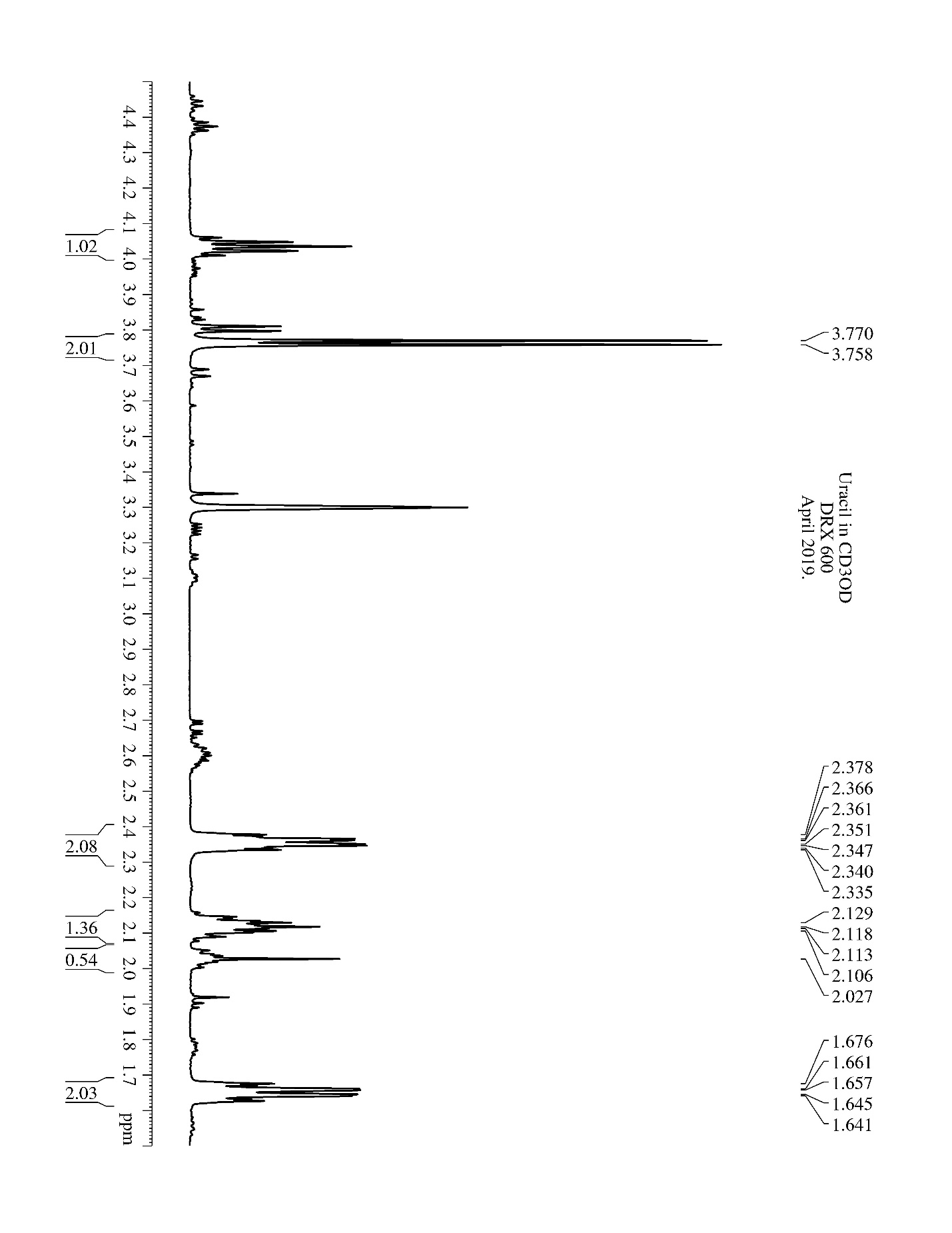


DRX 600 1D ^1^H NMR Spectra of Uracil **15** in CD_3_OD


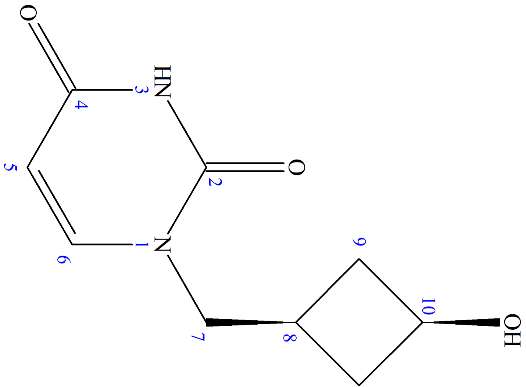

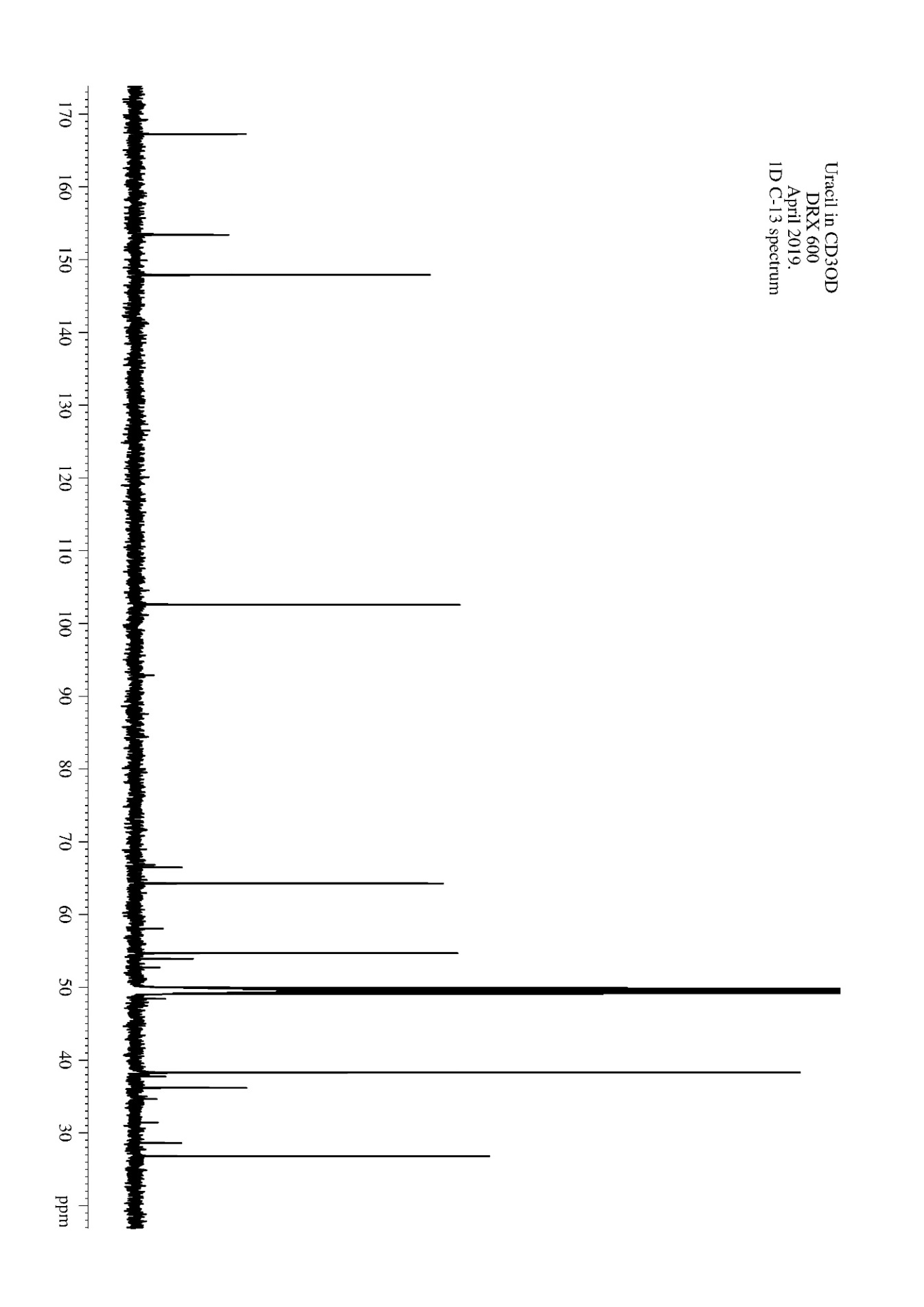


5

7

10

9

8

6

2

4

DRX 600 1D ^13^C NMR Spectra of Uracil **15** in CD_3_OD


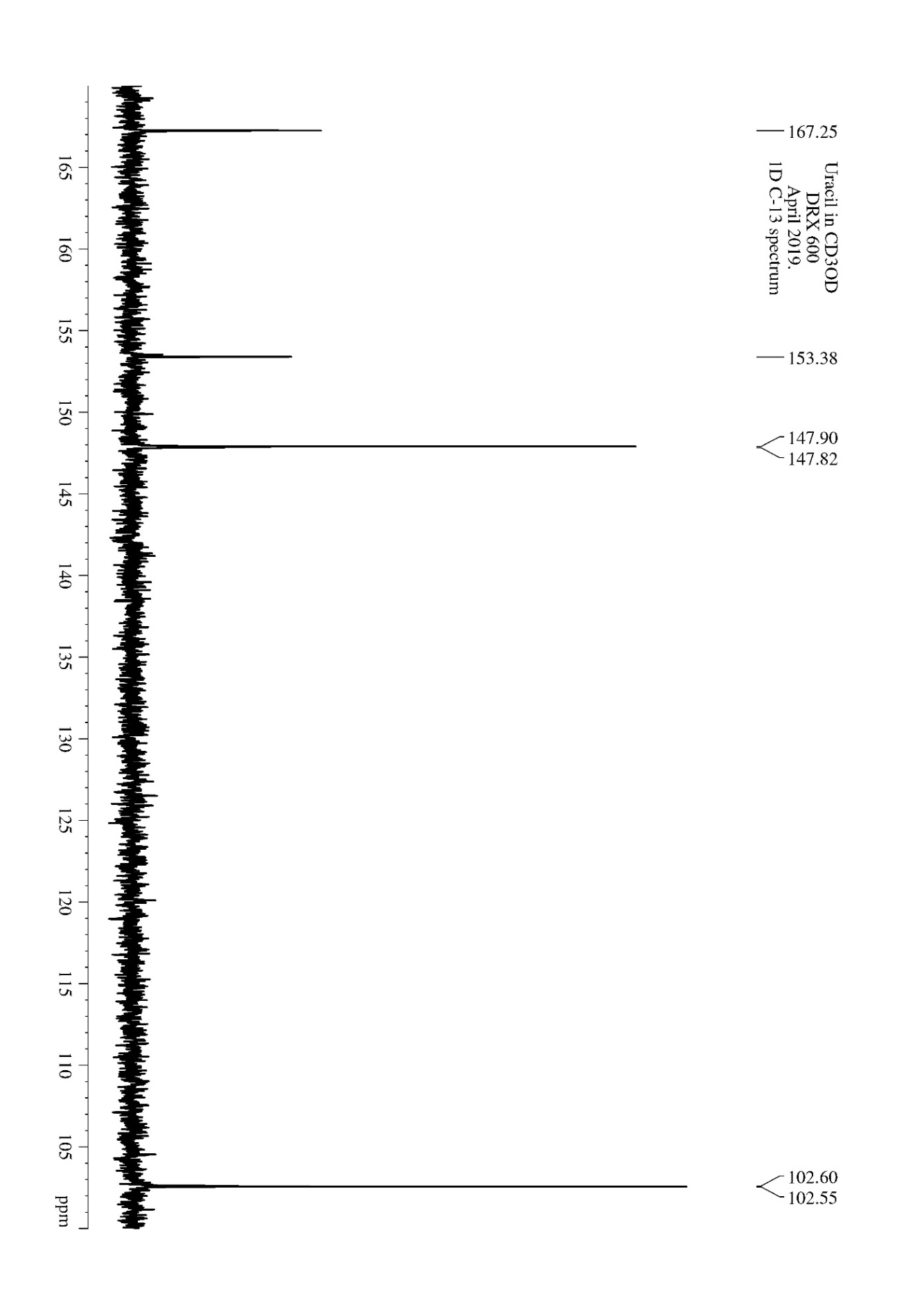


DRX 600 1D ^13^C NMR Spectra of Uracil **15** in CD_3_OD


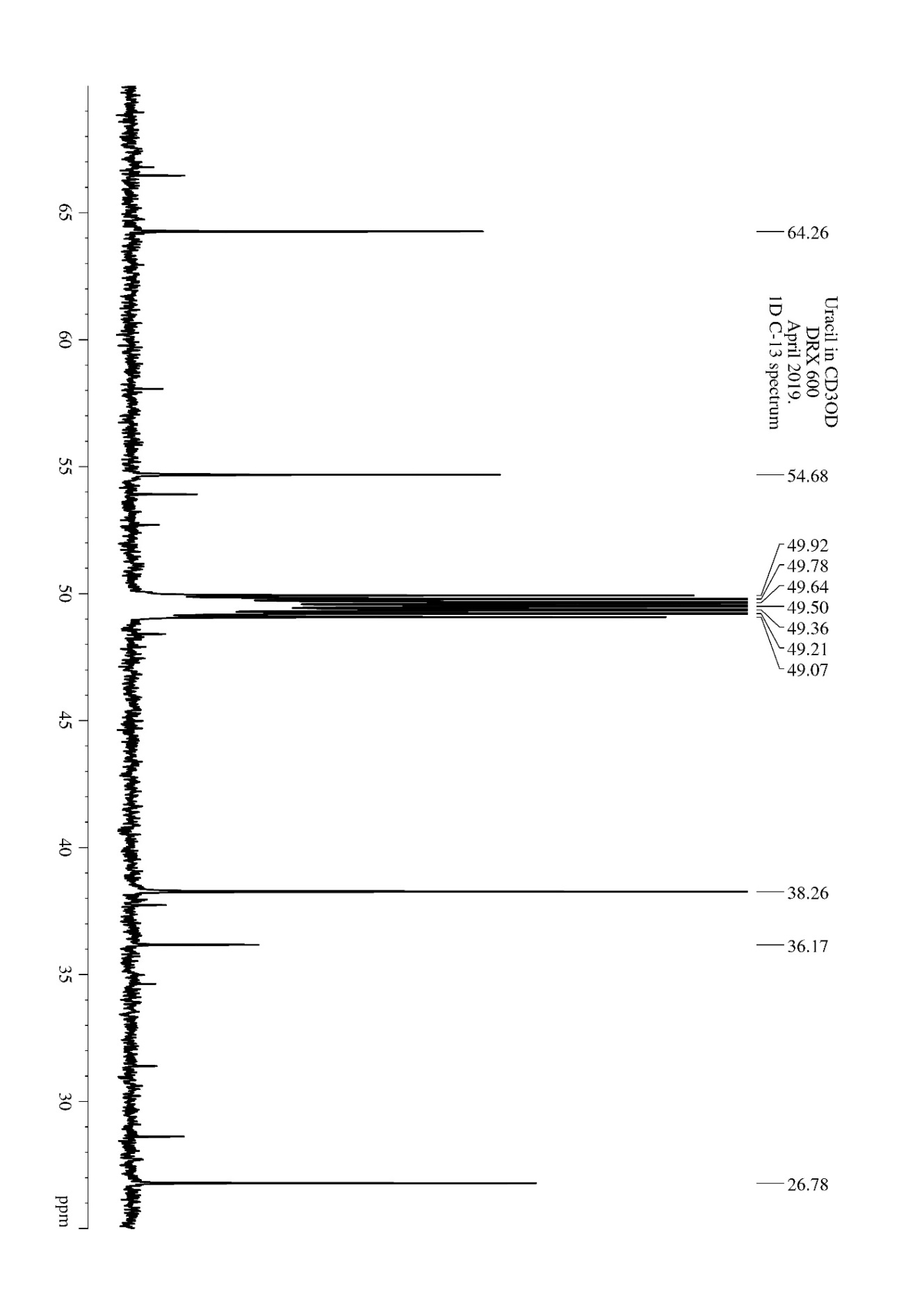


DRX 600 1D ^13^C NMR Spectra of Uracil **15** in CD_3_OD


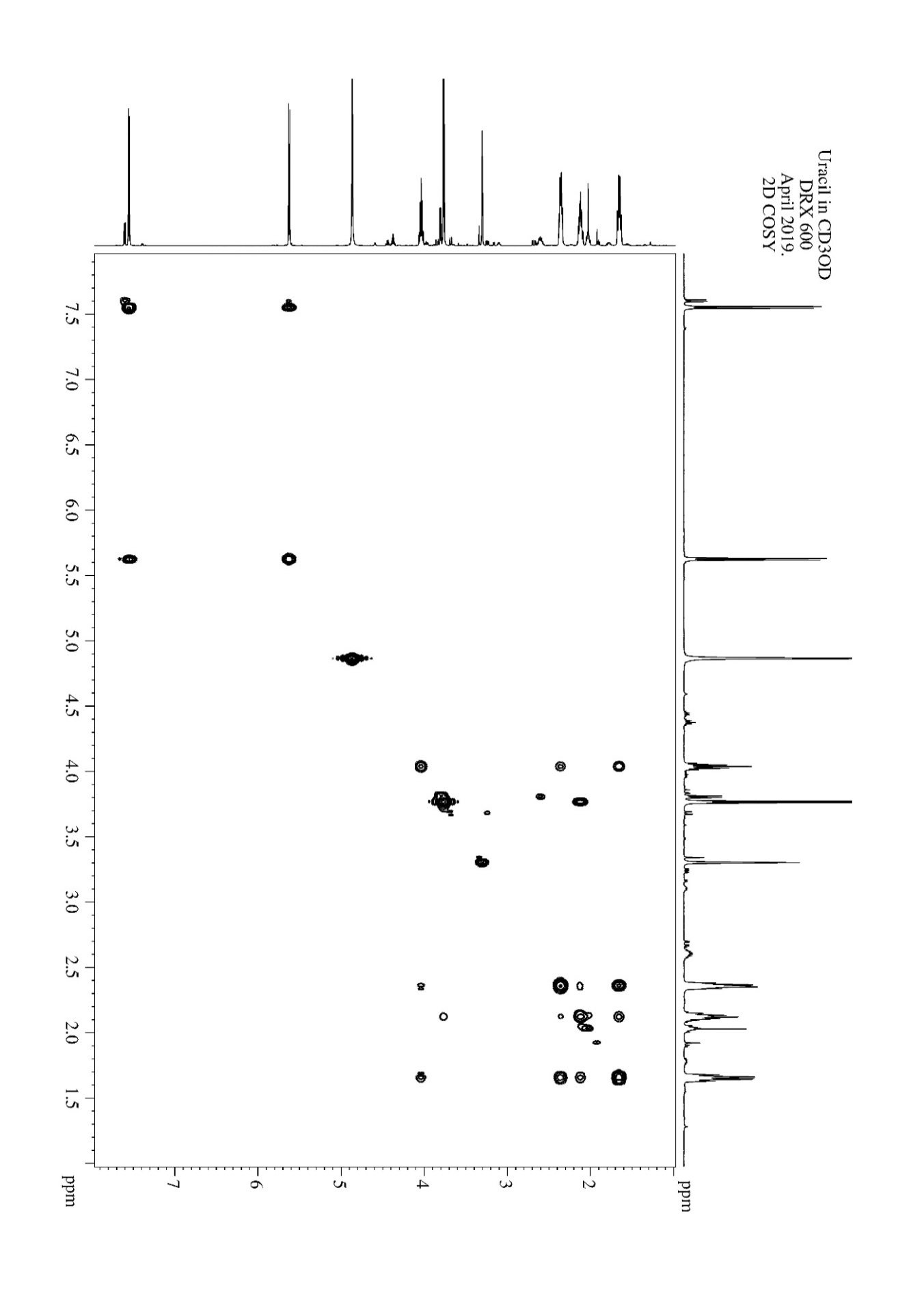


DRX 600 2D ^1^H-^1^H COSY NMR Spectra of Uracil **15** in CD_3_OD


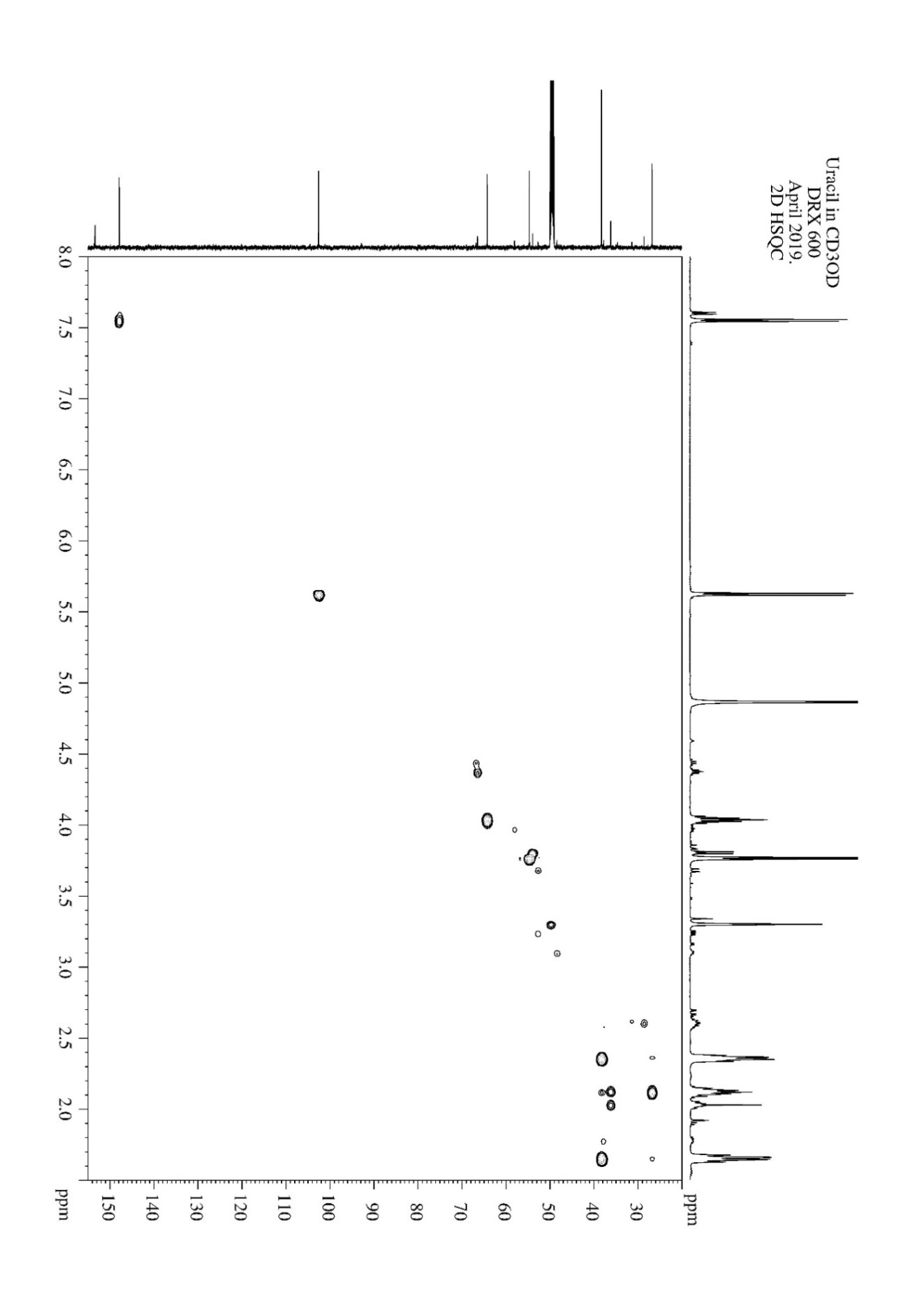


DRX 600 2D ^1^H-^13^C HSQC NMR Spectra of Uracil **15** in CD_3_OD


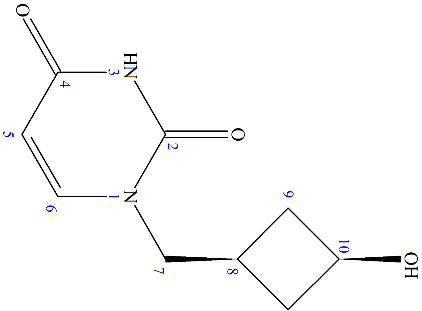

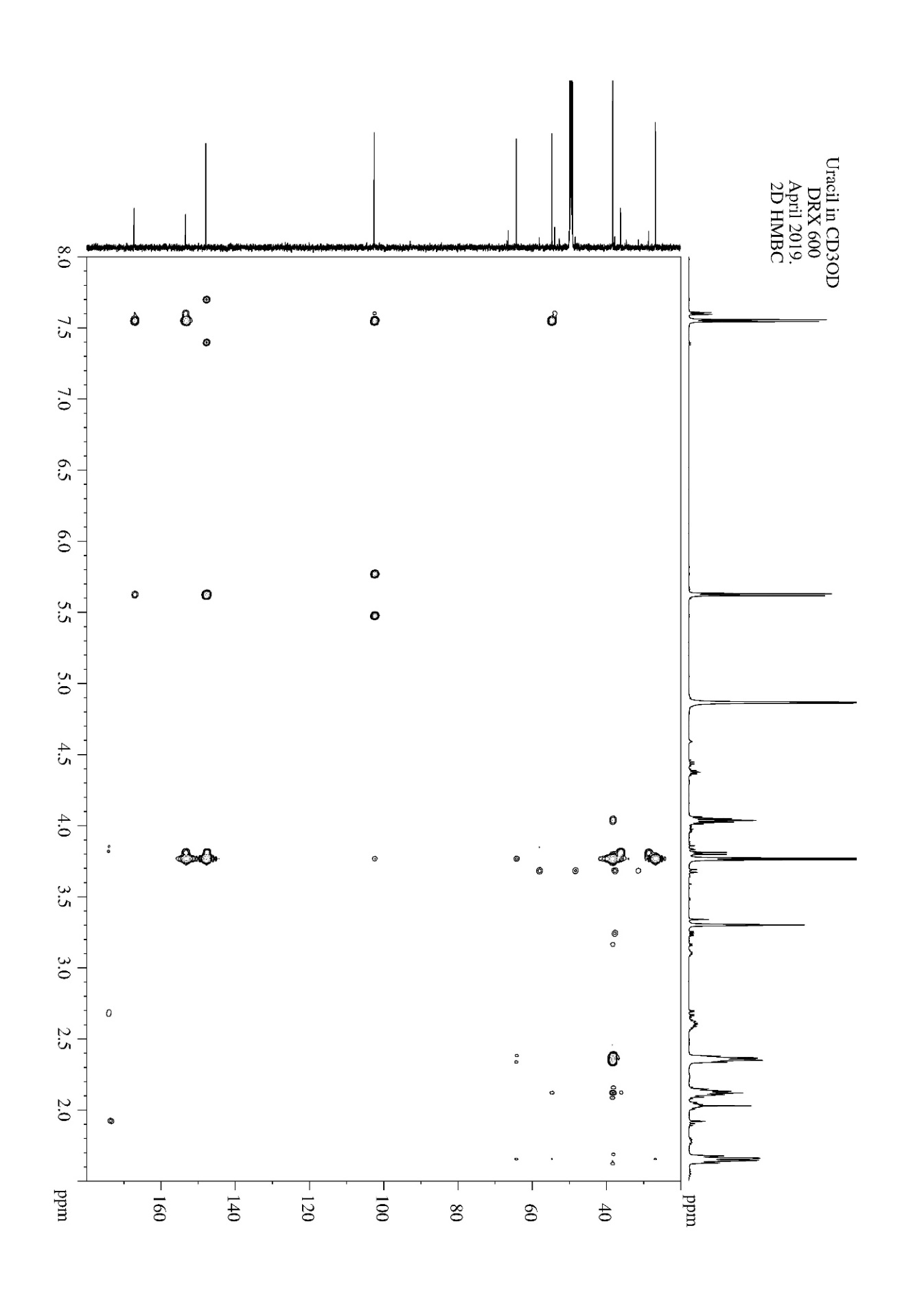


7-6

6-7

DRX 600 2D ^1^H-^13^C HMBC NMR Spectra of Uracil **15** in CD_3_OD


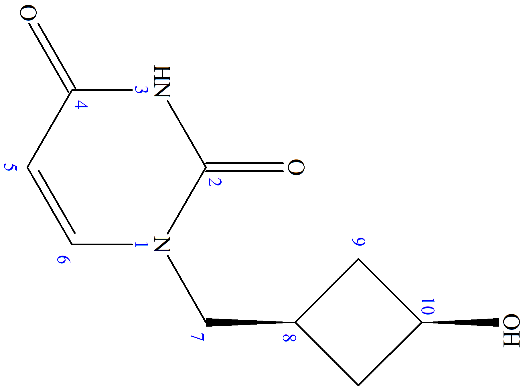

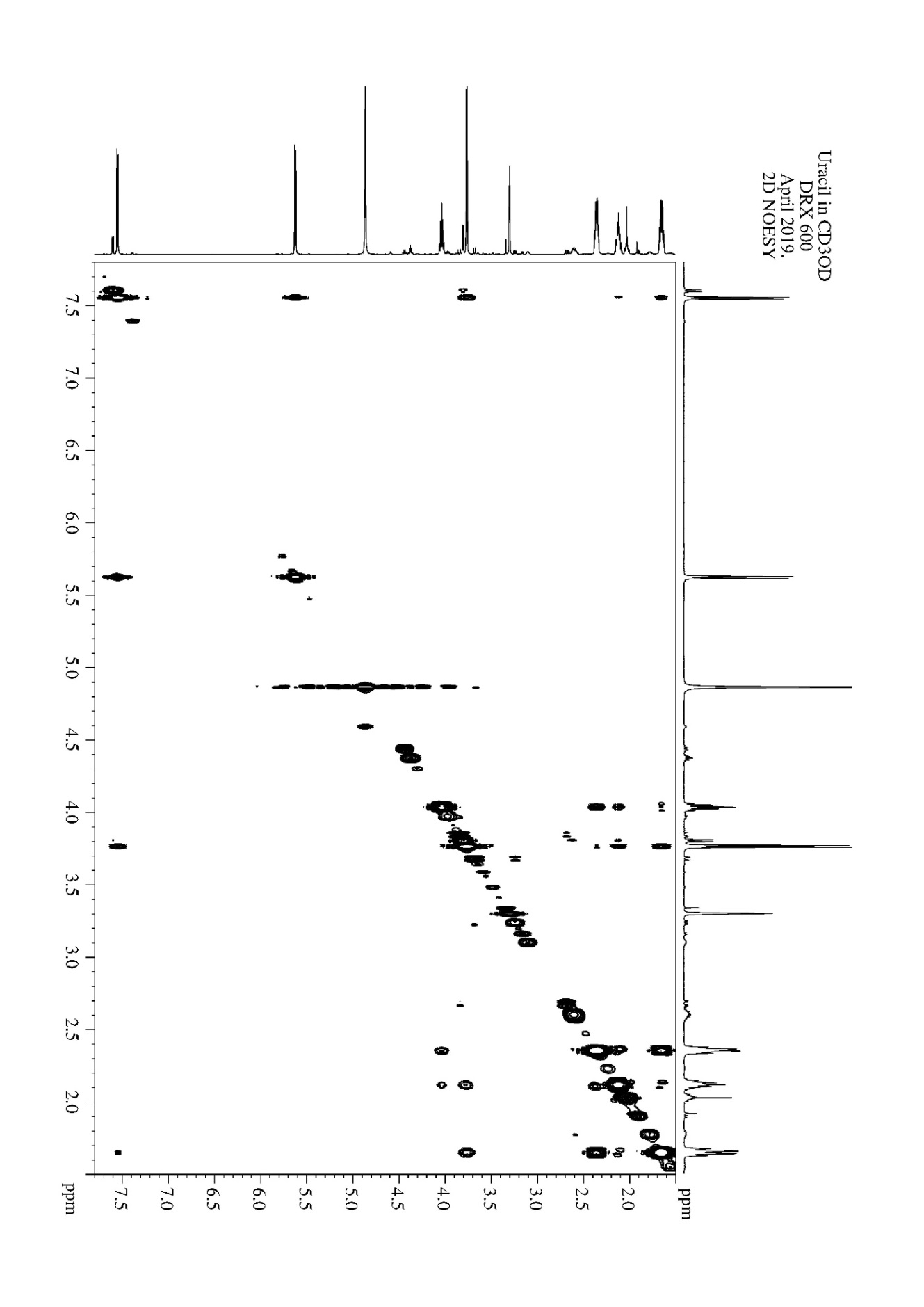


9 up

9 down

6-9

6-8

6-7

DRX 600 2D ^1^H-^1^H NOESY NMR Spectra of Uracil **15** in CD_3_OD


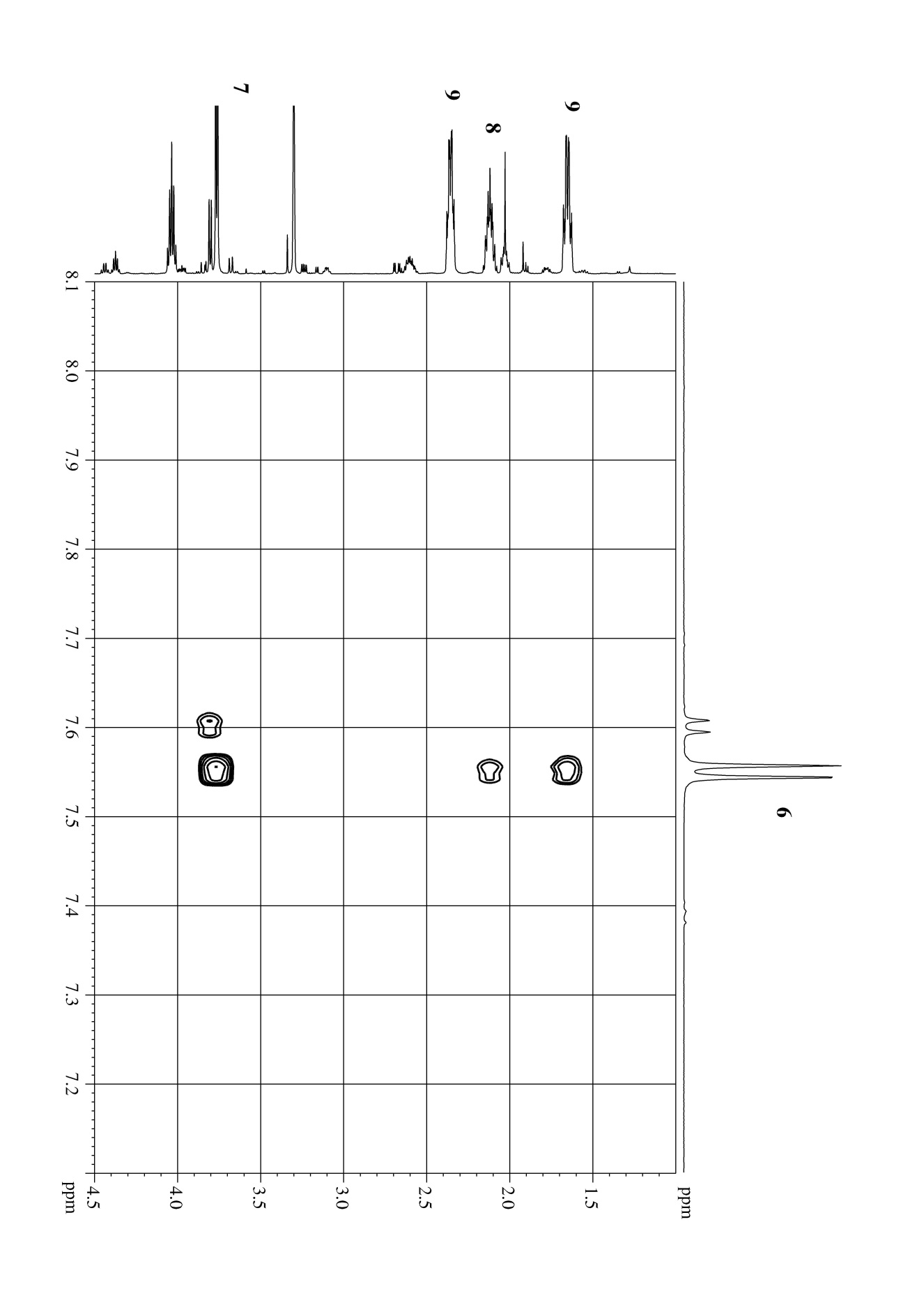


DRX 600 2D ^1^H-^1^H NOESY NMR Spectra of Uracil **15** in CD_3_OD


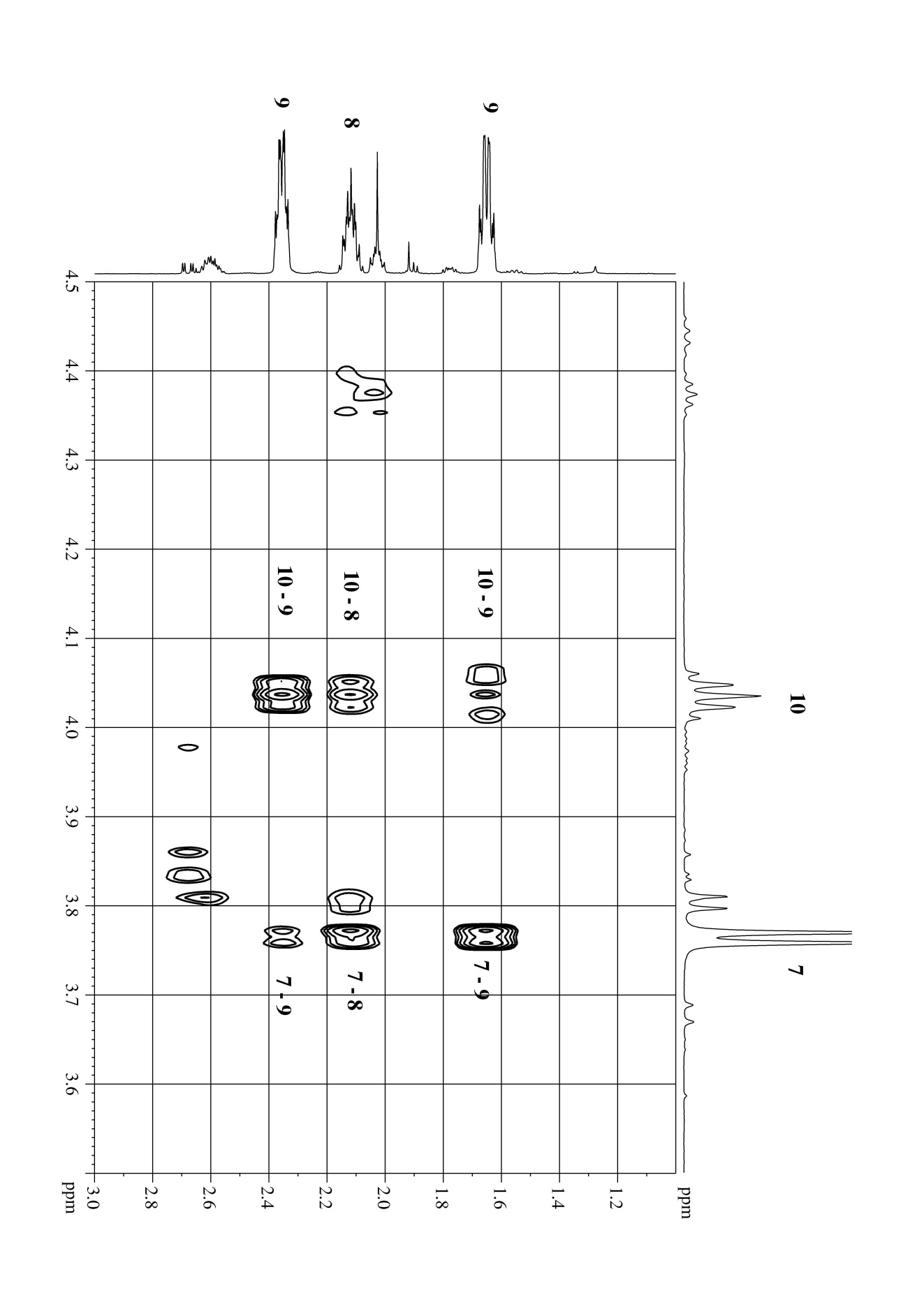


DRX 600 2D ^1^H-^1^H NOESY NMR Spectra of Uracil **15** in CD_3_OD


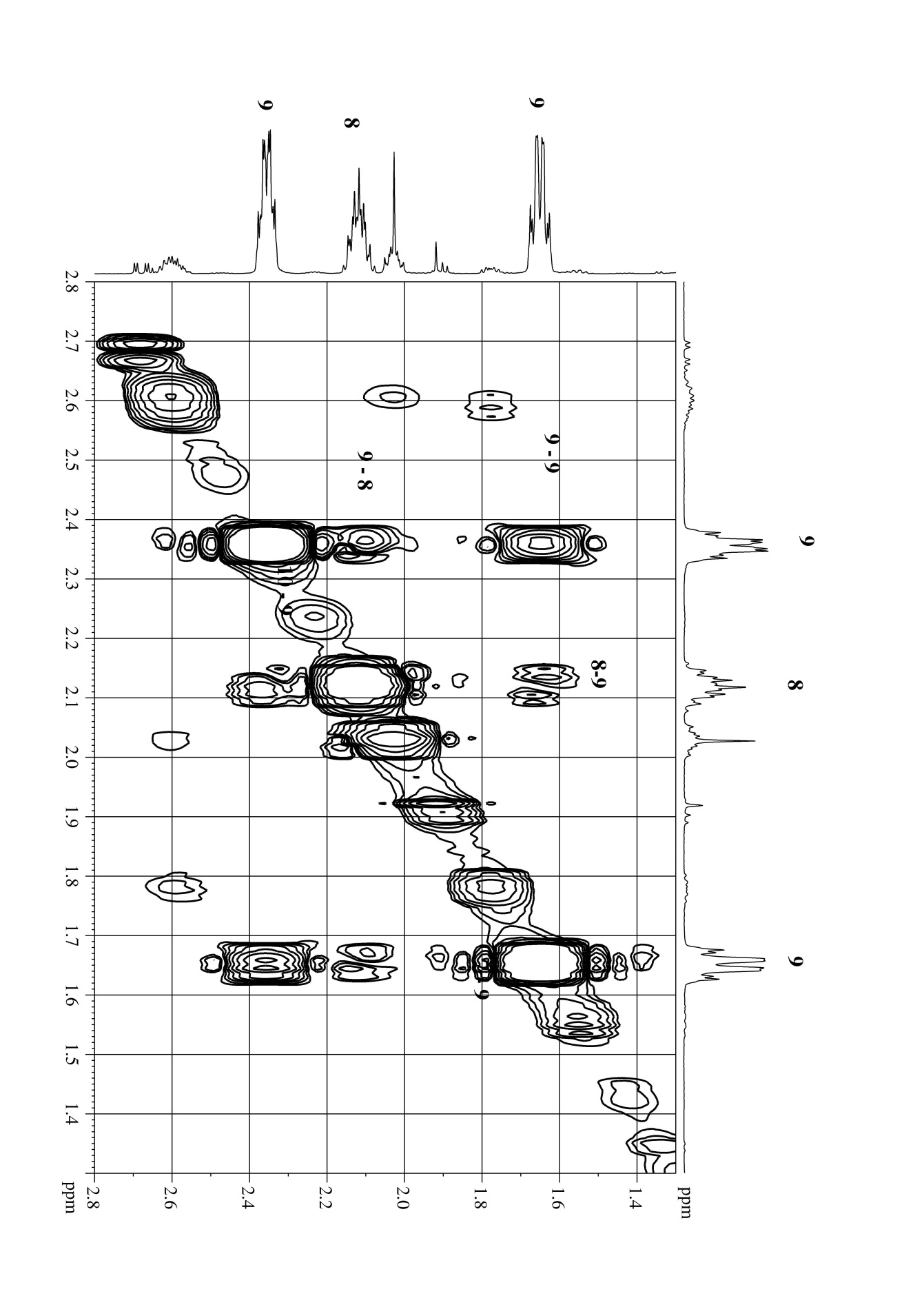


DRX 600 2D ^1^H-^1^H NOESY NMR Spectra of Uracil **15** in CD_3_OD

NMR Data for alcohol **18** in methanol


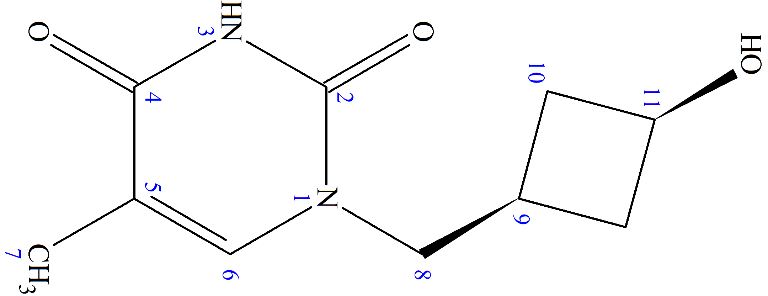

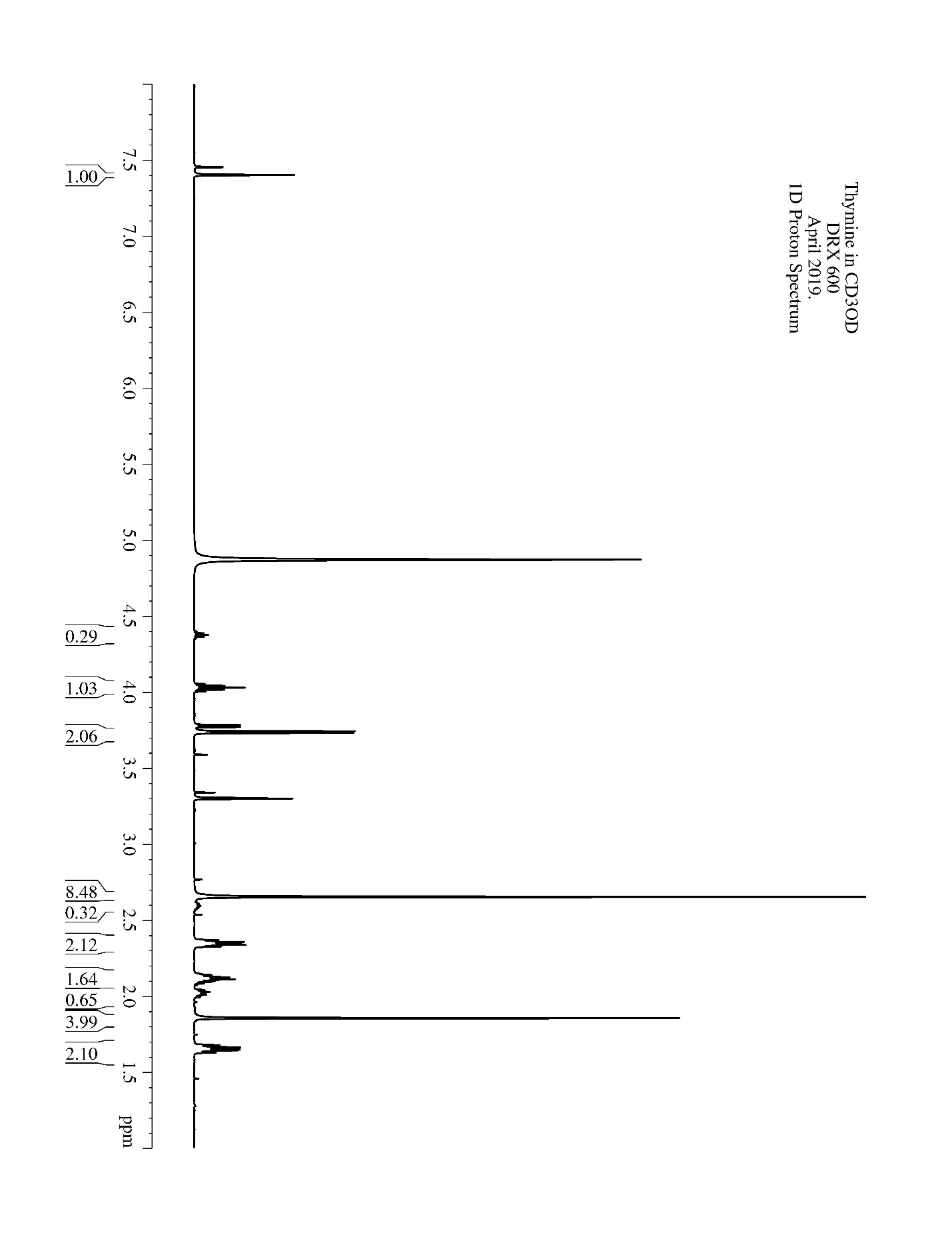


7

10

9

10

8

11

6


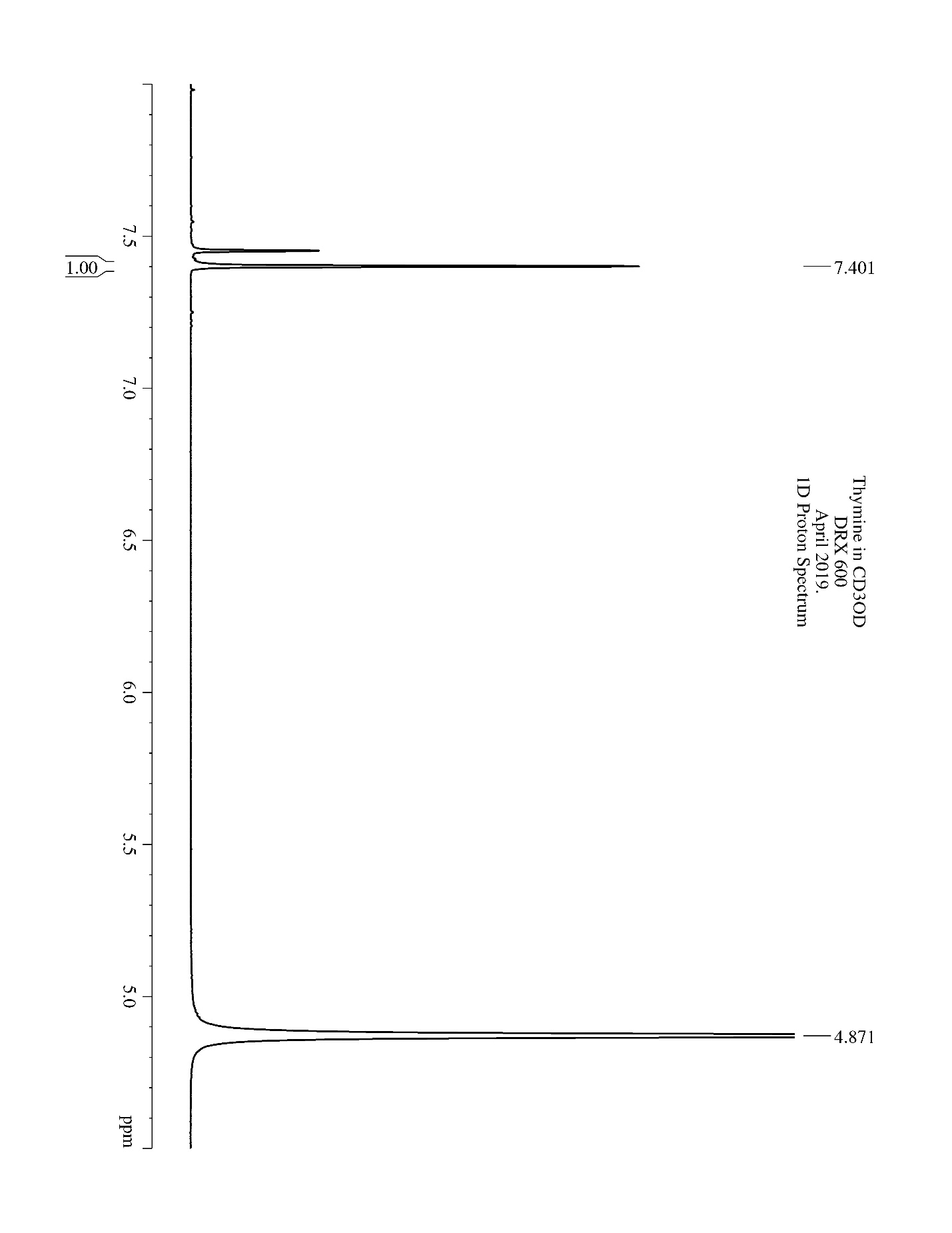


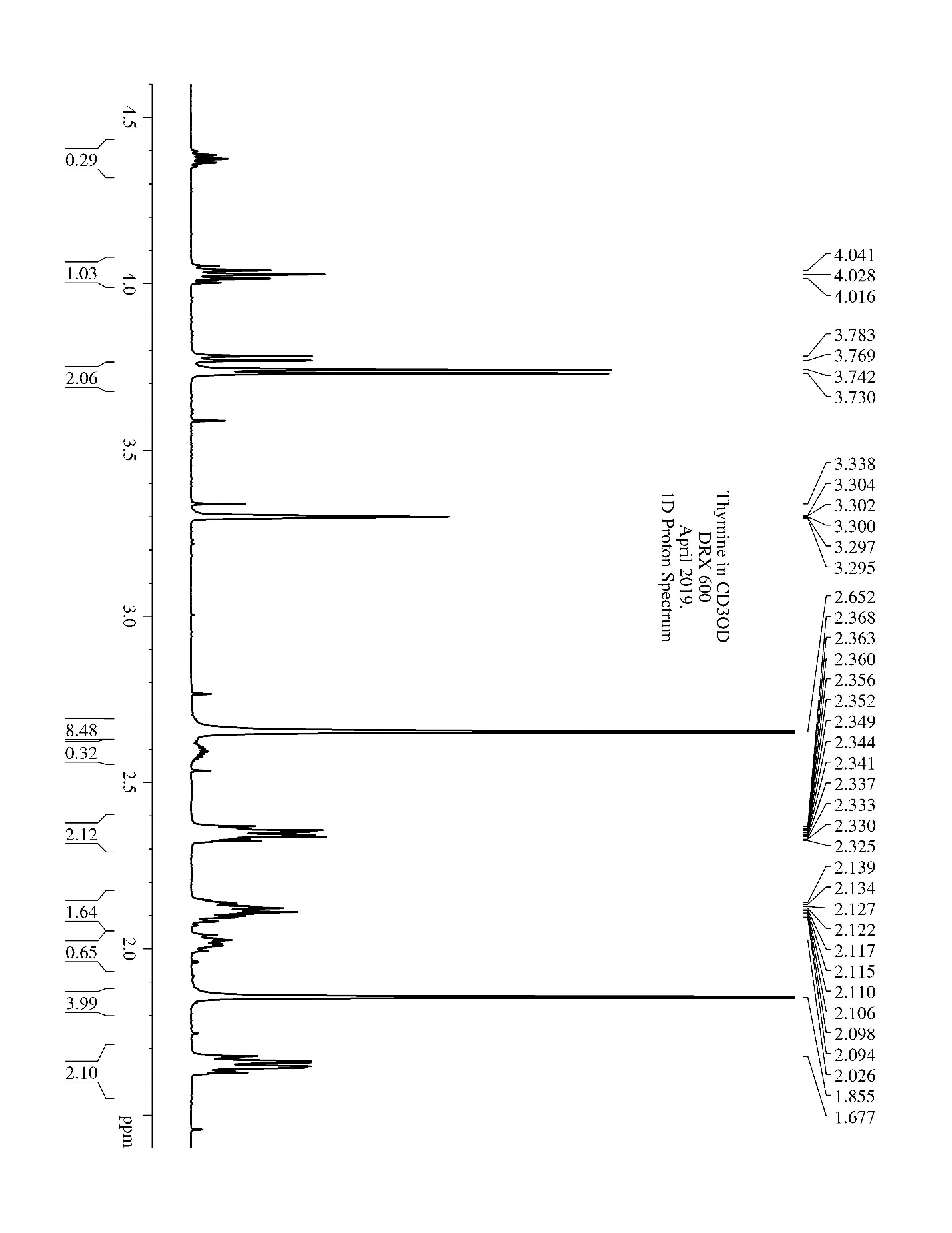


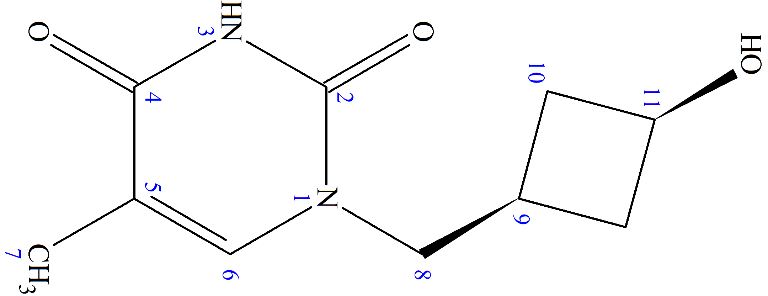

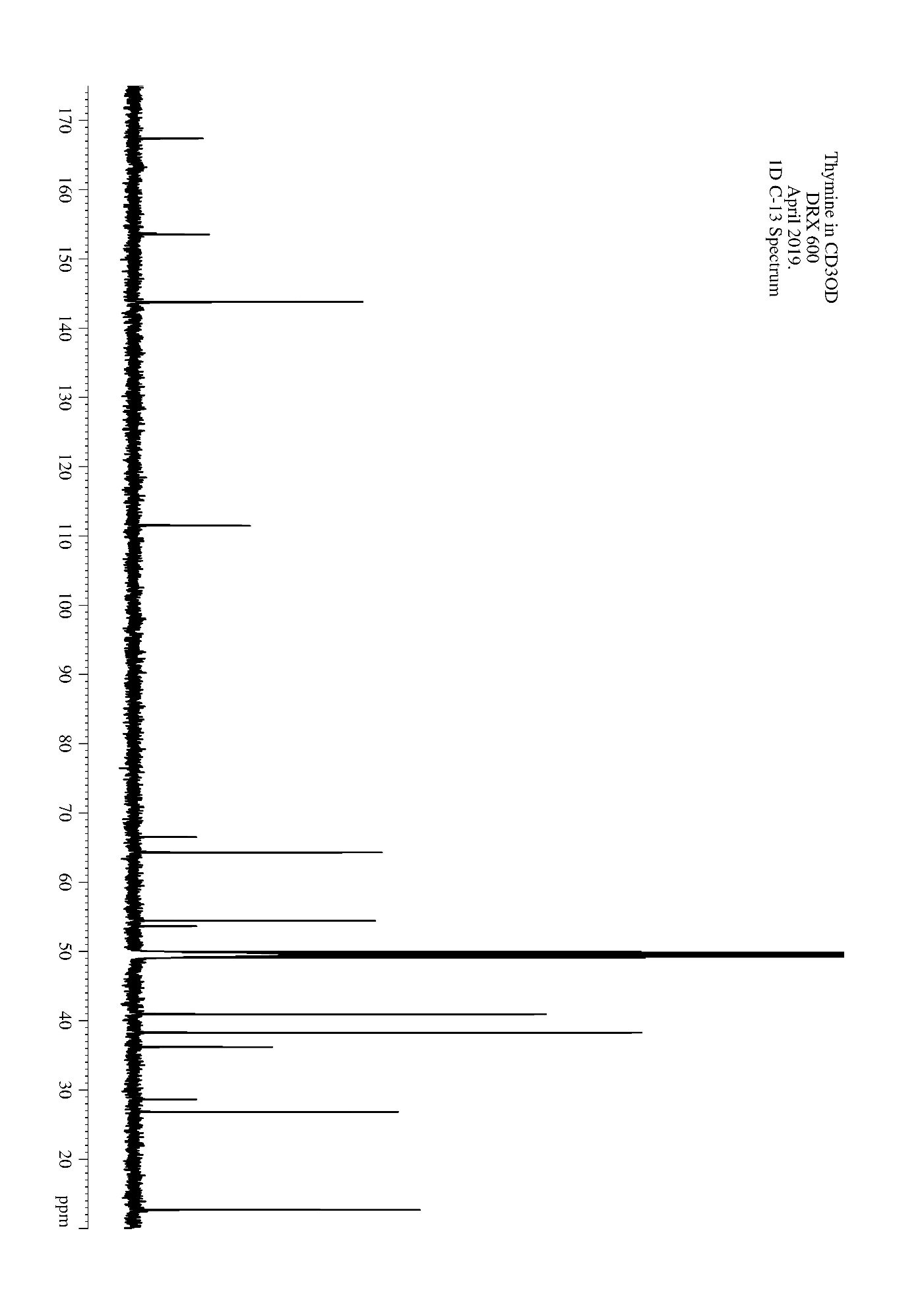


7

9

10

8

11

5

6

2

4


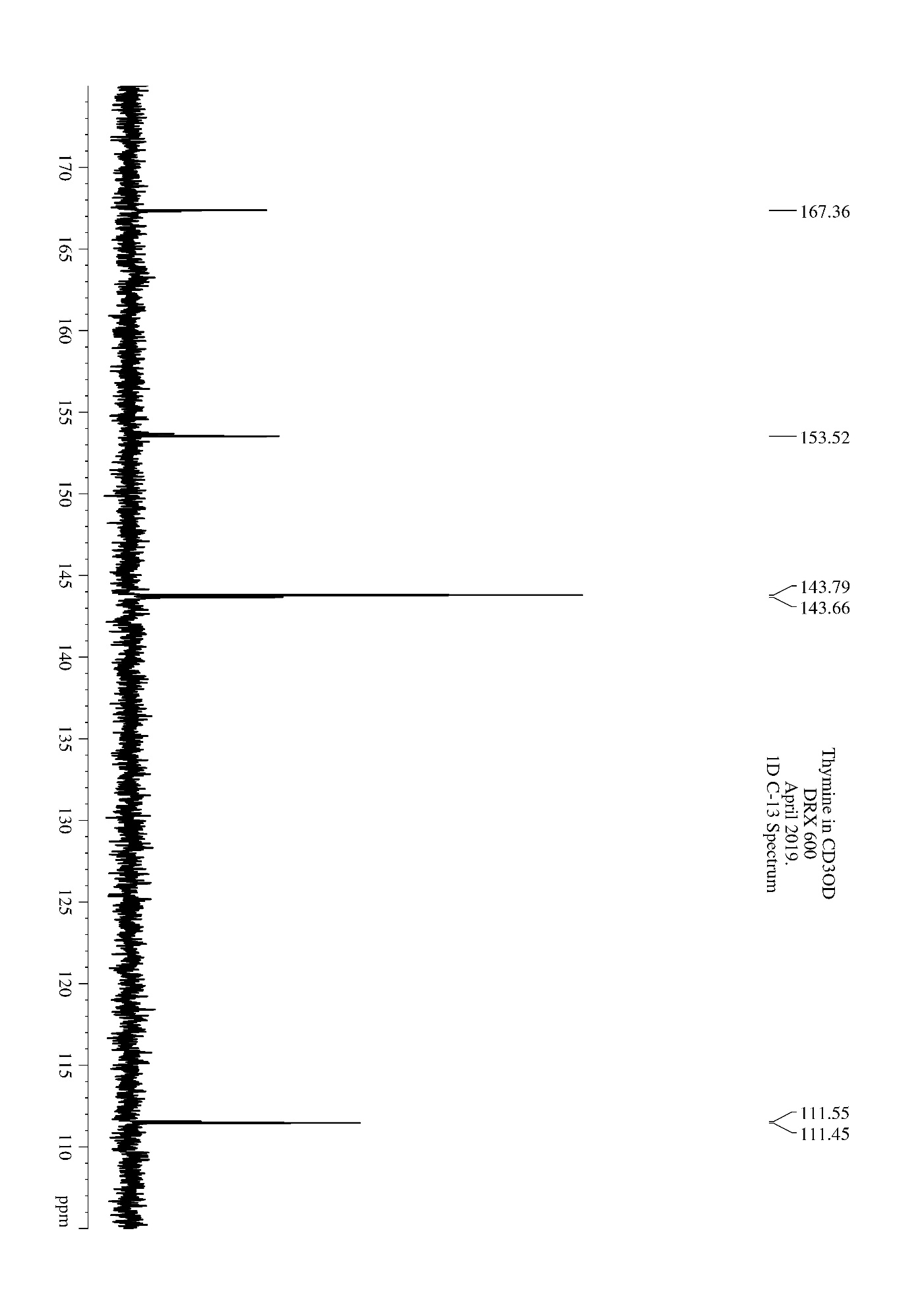


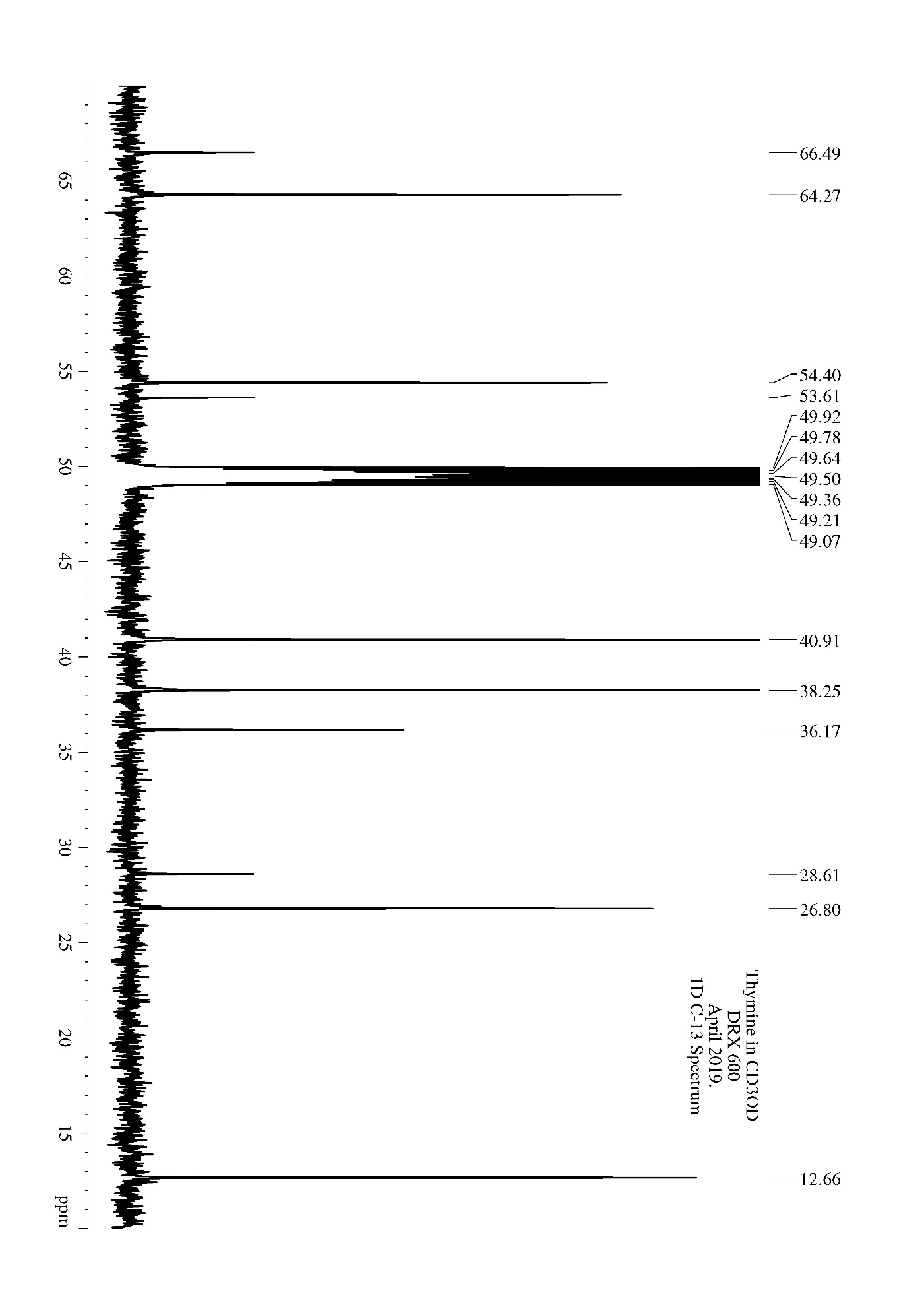


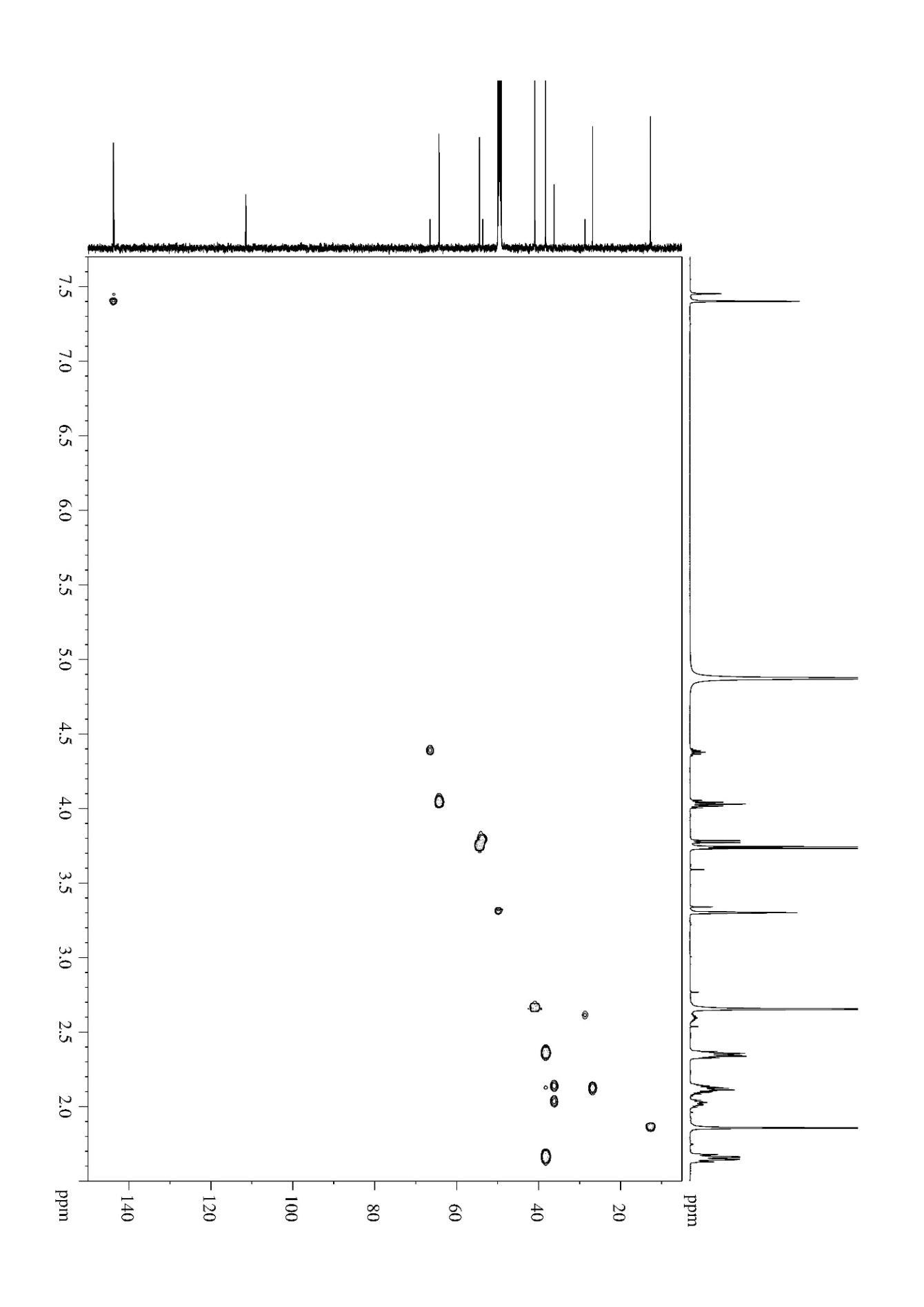


DRX 600 2D ^1^H-^13^C HSQC spectrum


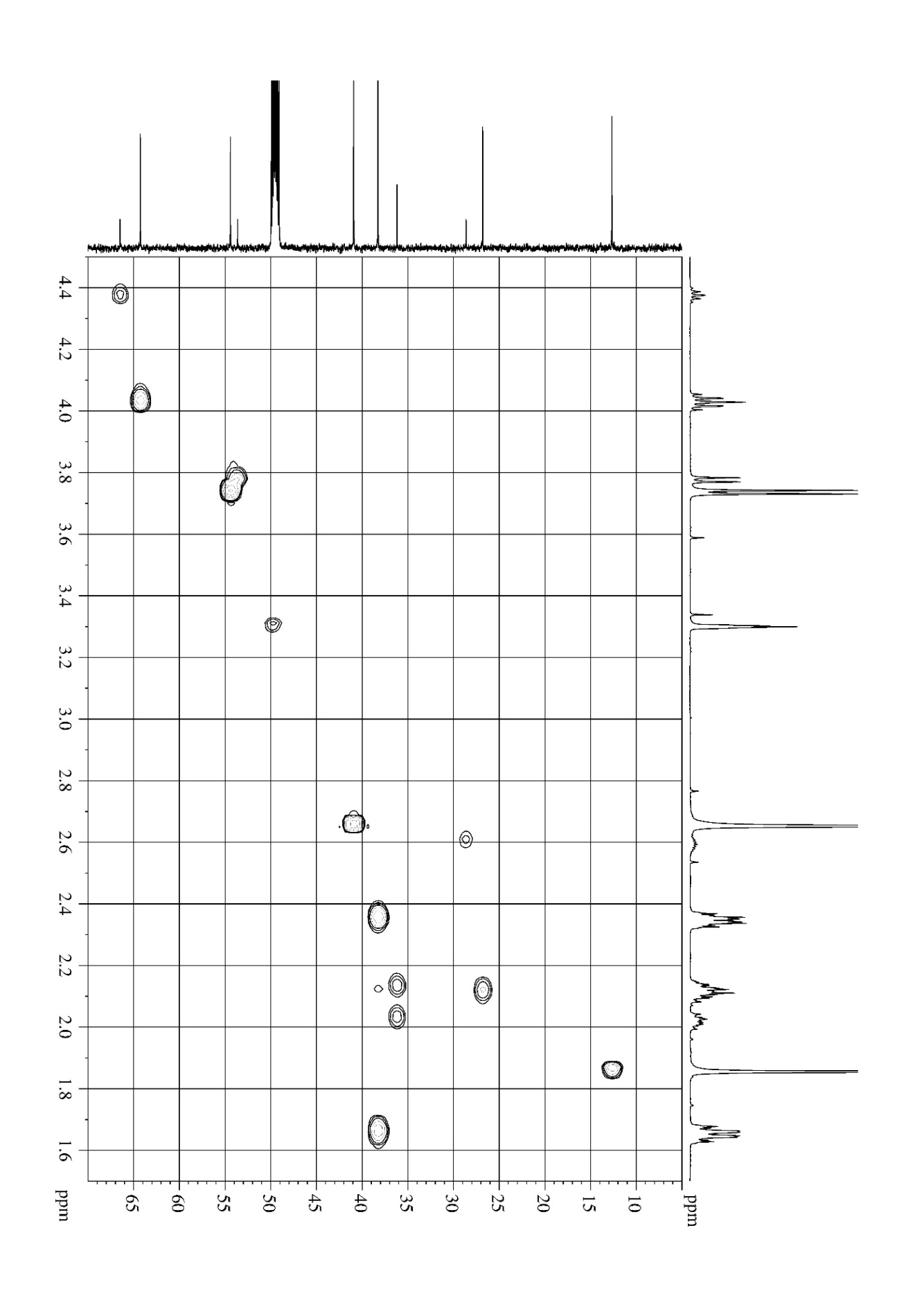


DRX 600 2D ^1^H-^13^C HSQC spectrum


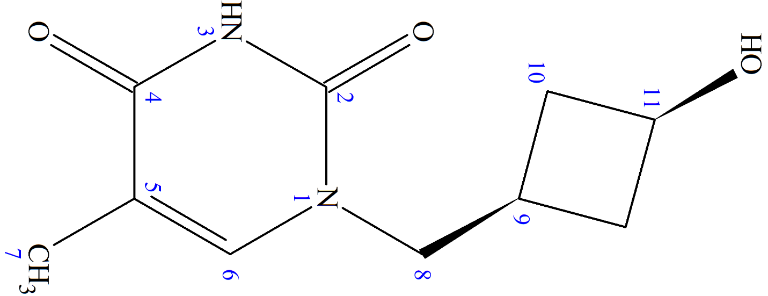

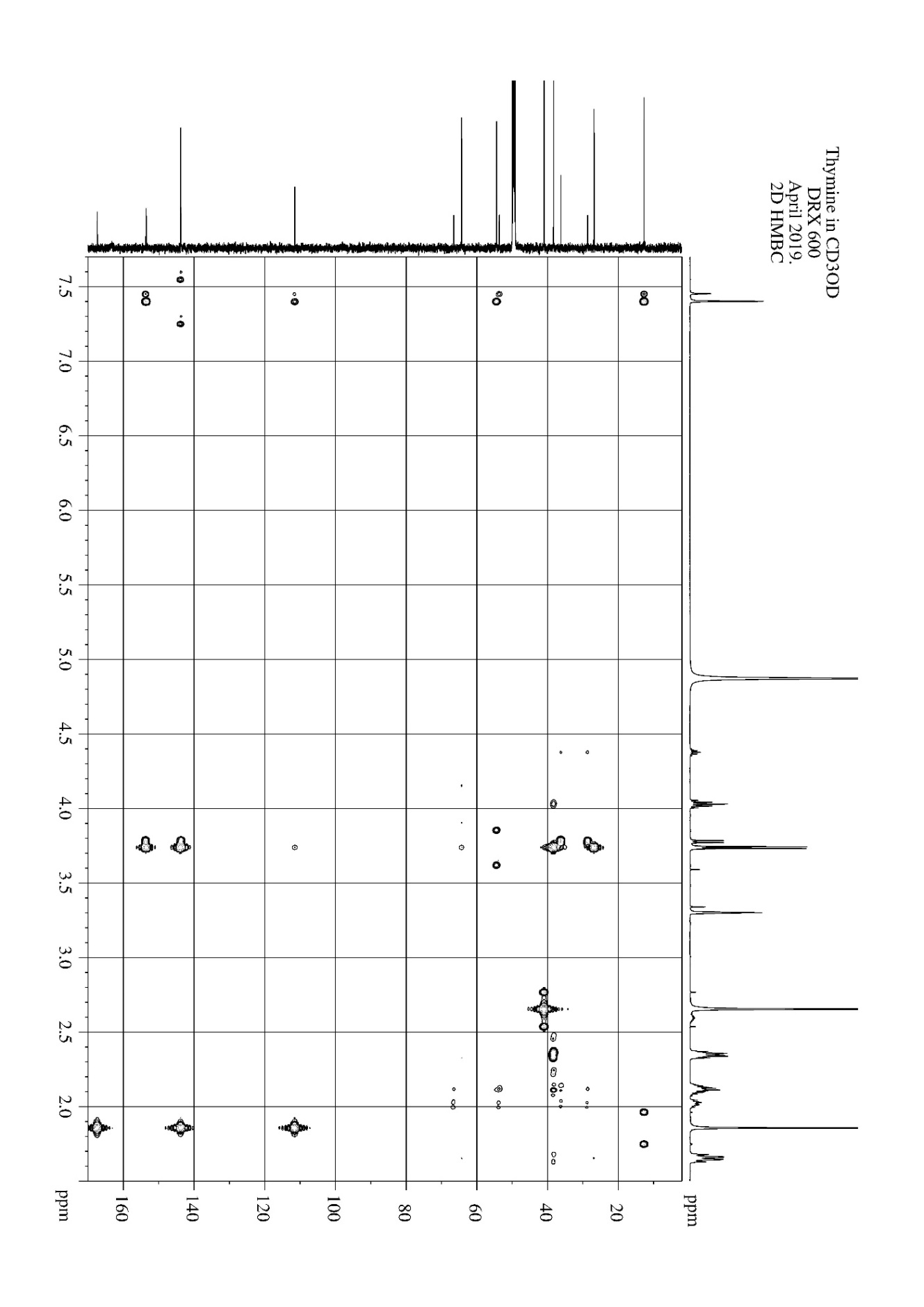


6-8

8-6

DRX 600 2D ^1^H-^13^C HMBC spectrum


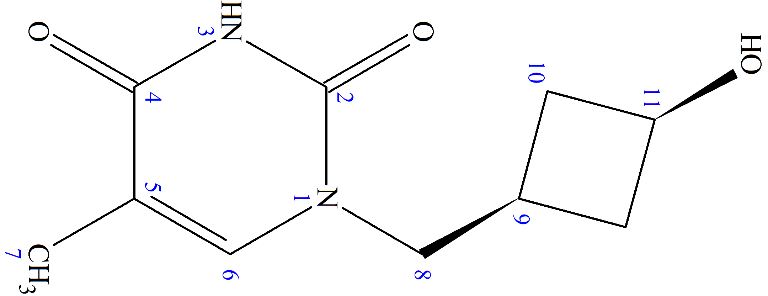

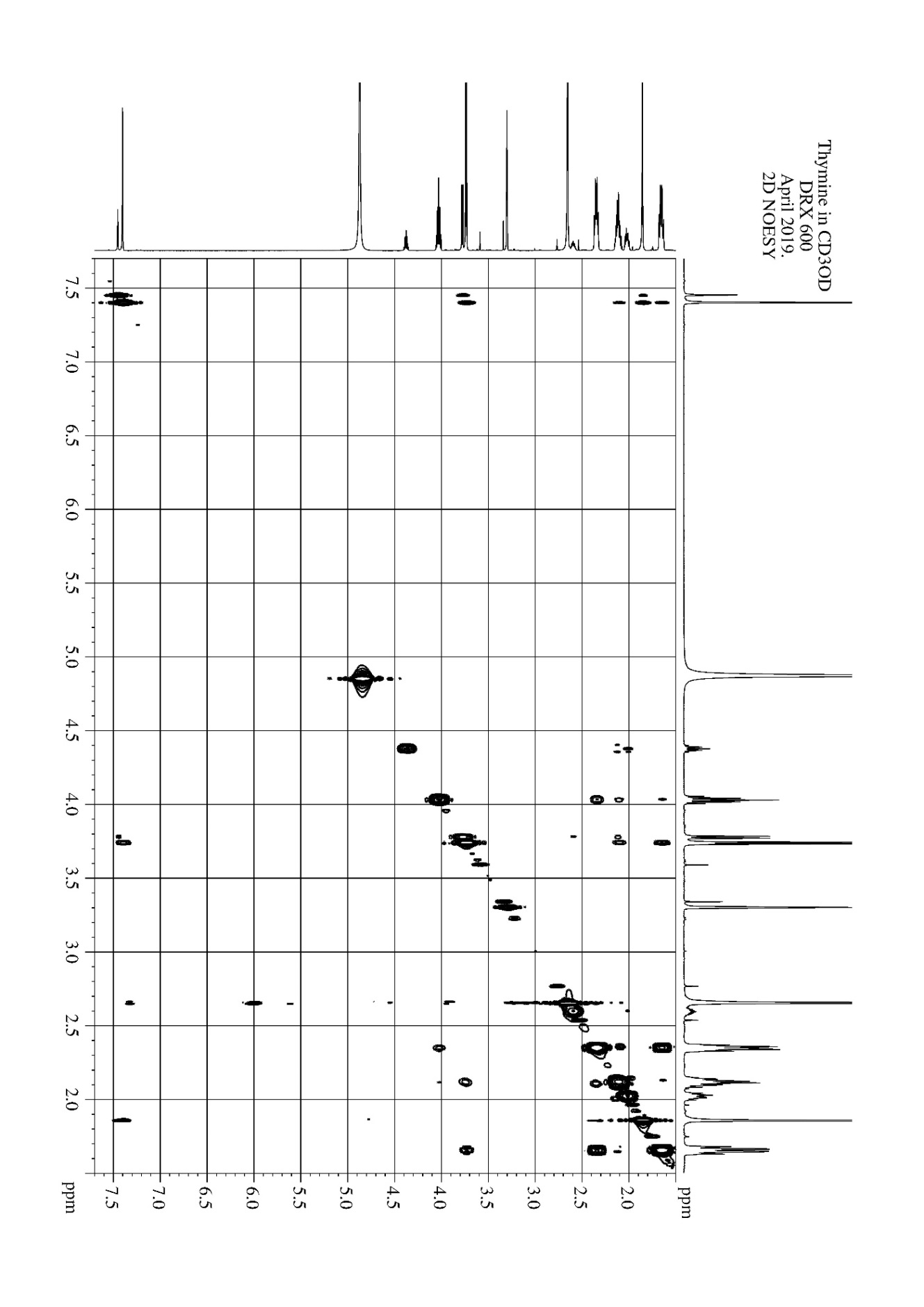


6-8

10 down

10 up

DRX 600 2D ^1^H-^1^H NOESY spectrum


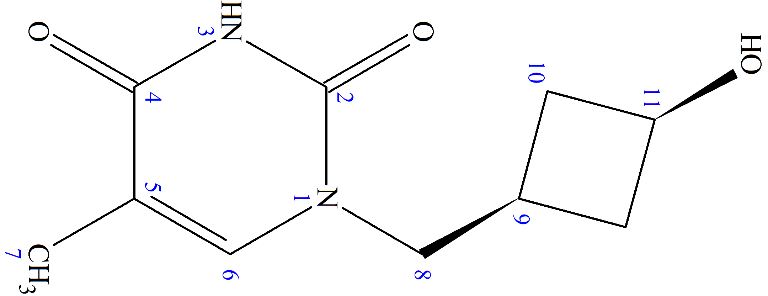

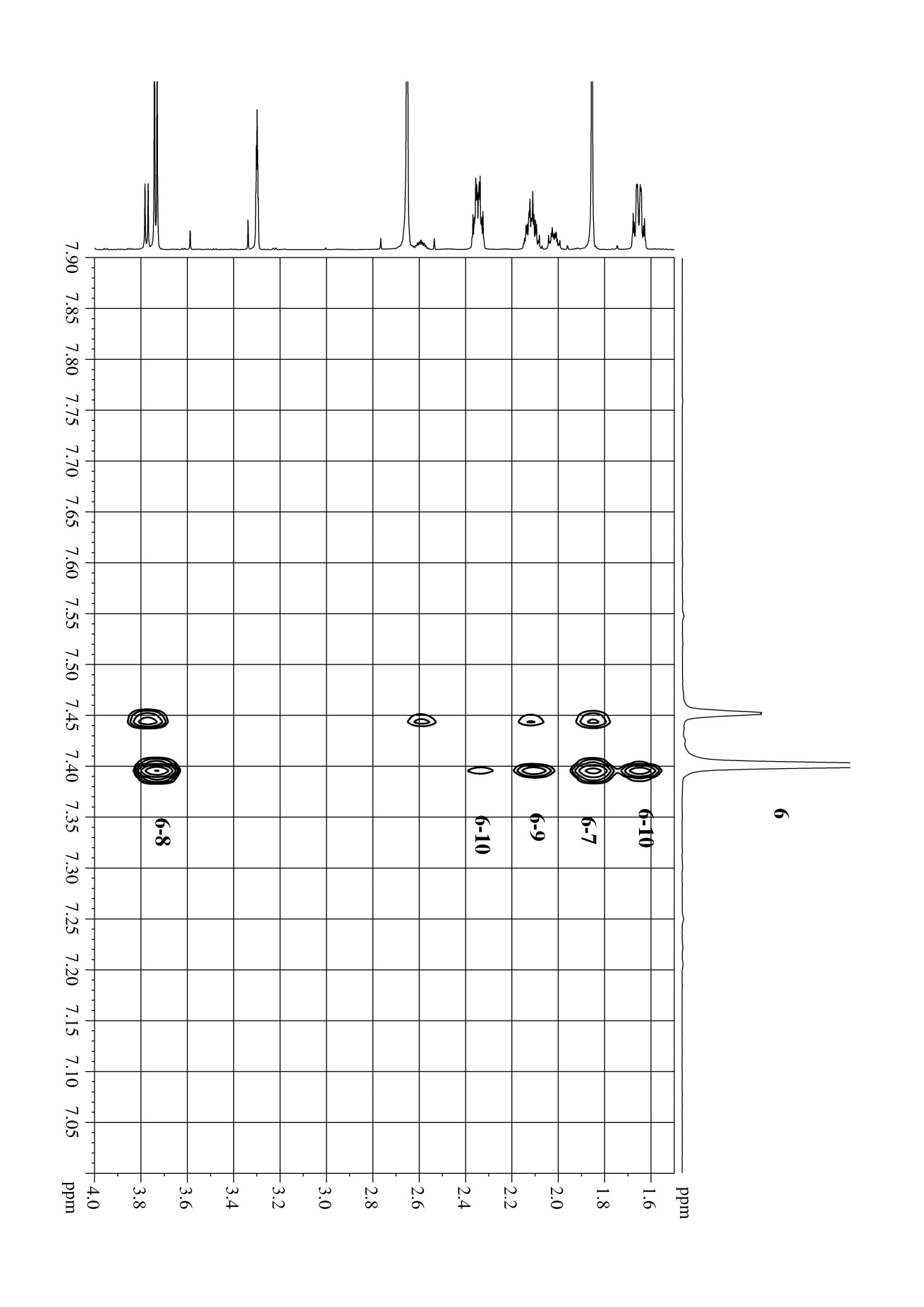


DRX 600 2D ^1^H-^1^H NOESY spectrum


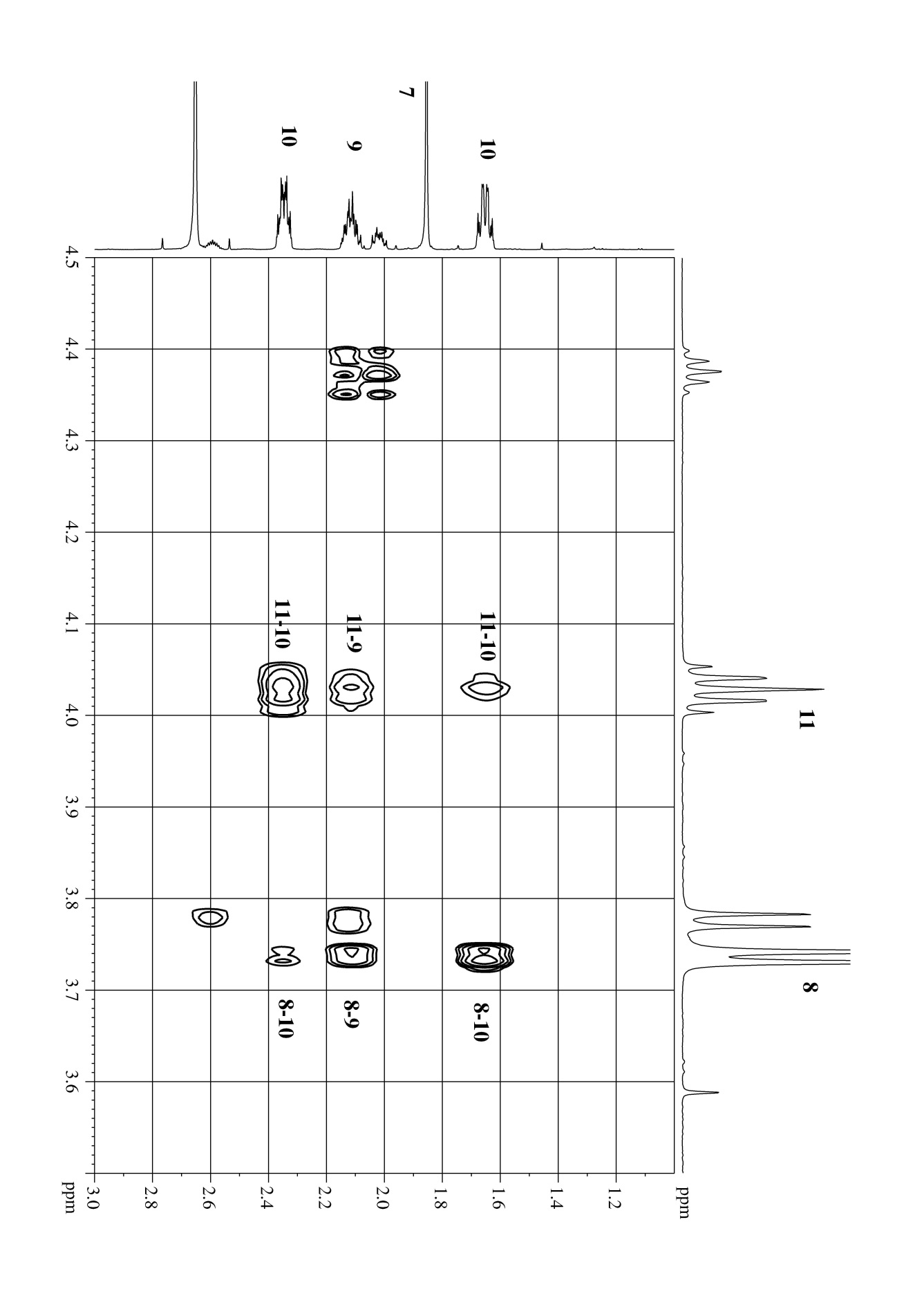


DRX 600 2D ^1^H-^1^H NOESY spectrum


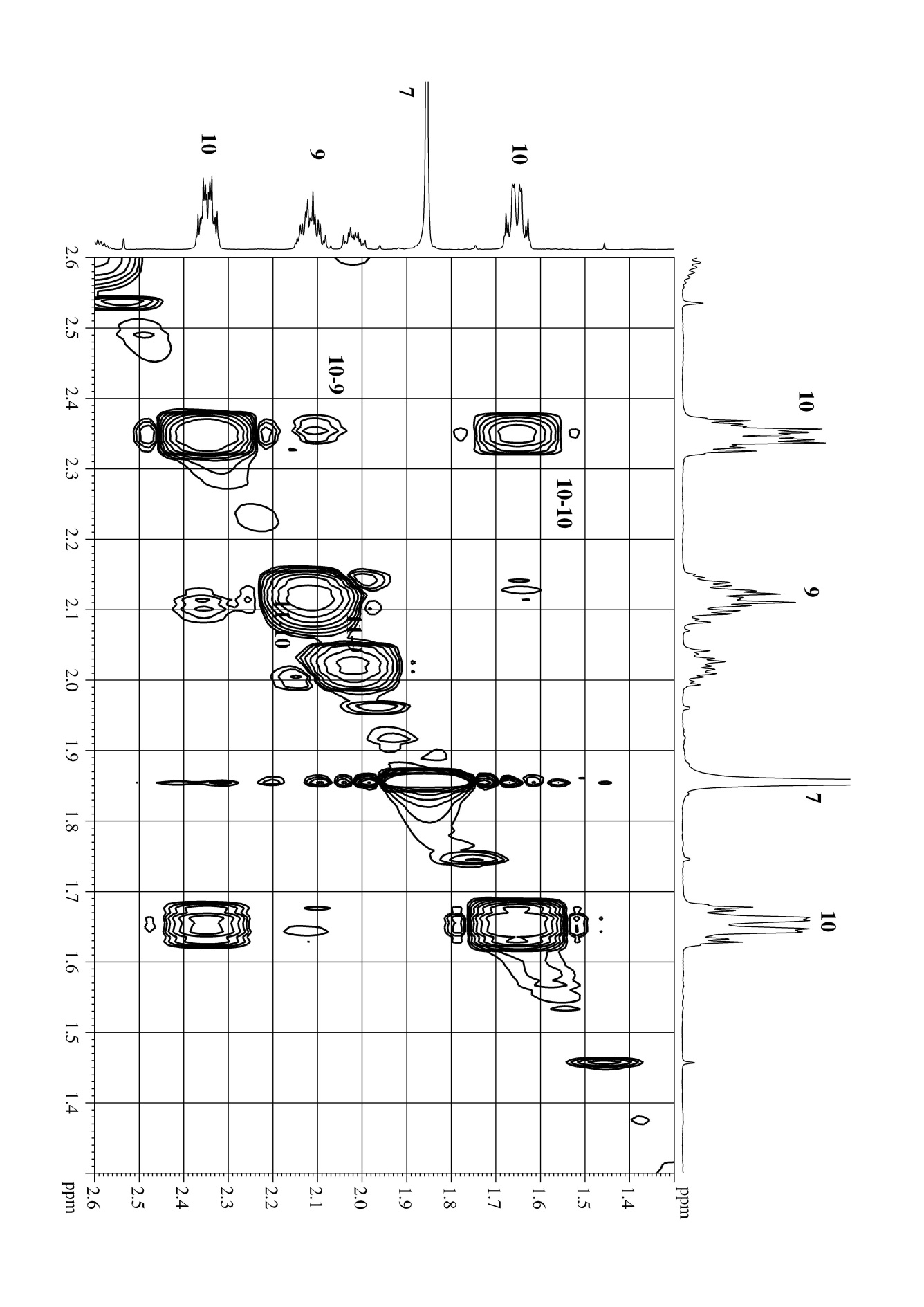


DRX 600 2D ^1^H-^1^H NOESY spectrum


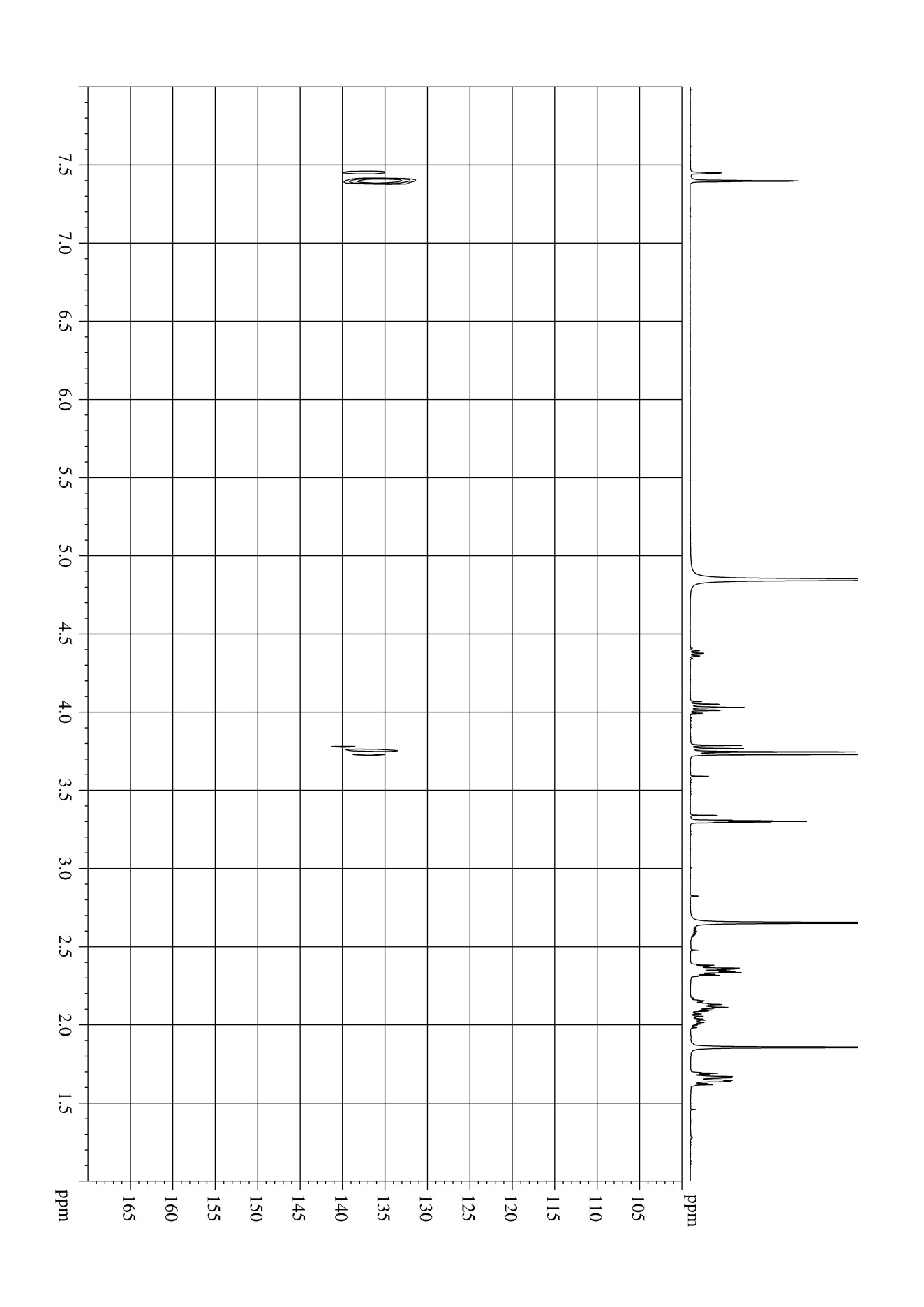


NEO 400 2D ^1^H-^15^N HMBC spectrum
